# How to control selectivity in alkane oxidation?†
†Electronic supplementary information (ESI) available. See DOI: 10.1039/c8sc04641g


**DOI:** 10.1039/c8sc04641g

**Published:** 2018-12-20

**Authors:** Xuan Li, Detre Teschner, Verena Streibel, Thomas Lunkenbein, Liudmyla Masliuk, Teng Fu, Yuanqing Wang, Travis Jones, Friedrich Seitz, Frank Girgsdies, Frank Rosowski, Robert Schlögl, Annette Trunschke

**Affiliations:** a Department of Inorganic Chemistry , Fritz-Haber-Institut der Max-Planck-Gesellschaft , Faradayweg 4-6 , 14195 Berlin , Germany . Email: trunschke@fhi-berlin.mpg.de ; Tel: +49 30 8413 4457; b UniCat-BASF Joint Lab , Technische Universität Berlin , Sekr. EW K 01, Hardenbergstraße 36 , 10623 Berlin , Germany; c Department of Heterogeneous Reactions , Max-Planck-Institut für Chemische Energiekonversion , Stiftstraße 34-36 , 45470 Mülheim a. d. Ruhr , Germany; d BASF SE , Process Research and Chemical Engineering , Heterogeneous Catalysis , Carl-Bosch-Straße 38 , 67056 Ludwigshafen , Germany

## Abstract

The well-defined particle morphology of crystalline MnWO_4_ catalysts investigated in the present study facilitates obtaining insight into the origin of selectivity limitations in alkane oxidation. Hydrothermal synthesis at variable pH values granted access to a series of phase-pure MnWO_4_ catalysts with particles ranging from cube-like (aspect ratio 1.5) to rod- or needle-like (aspect ratio 6.8) shapes. Kinetic studies reveal a strong dependence of the propane consumption rate on the particle shape. The true origin of the structure sensitivity was unraveled by comprehensive bulk and surface analysis using nitrogen adsorption, XRD, SEM, ADF-STEM, STEM-EELS, XPS, multi-laser excitation Raman and DRIFT/operando FTIR spectroscopies, temperature-programmed oxidation (TPO), *in situ* NEXAFS, and DFT calculations. The active phase is composed of a thin manganese oxy-hydroxide layer formed on the surface of crystalline MnWO_4._ The differences in catalytic performance within the series clearly illustrate that the structural motif as the most popular descriptor in oxidation catalysis is not essential, since all MnWO_4_ catalysts in the series under study exhibit the same bulk crystal structure and bulk chemical composition and are phase pure and homogenous. The variable particle shape serves as a proxy that reflects the formation of varying abundance of redox active Mn^2+^/Mn^3+^ surface sites, which correlates with catalytic activity. Operando FTIR spectroscopy directly confirms the formation of Mn–OH surface species by abstraction of hydrogen atoms from the propane molecule on nucleophilic oxygen atoms and suggests that active site regeneration occurs *via* oxidative dehydrogenation of Mn–OH species indicating a single-site nature of the active sites that does not allow four-electron reduction of molecular oxygen. Instead, intermediates are created that cause side reactions and lower the selectivity. The findings highlight fundamental design criteria that may be applied to advance the development of new alkane oxidation catalysts with improved selectivity.

## Abstract 2

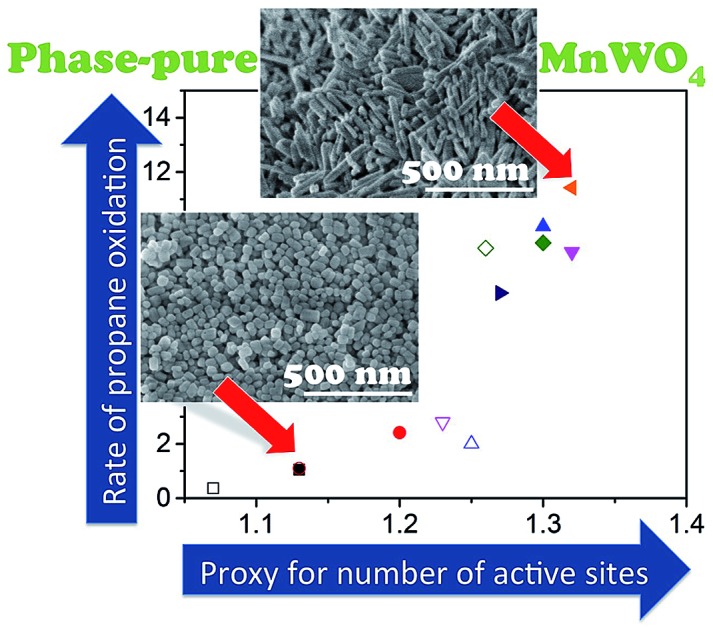
The bulk crystal structure of an oxidation catalyst as the most popular descriptor in oxidation catalysis is not solely responsible for catalytic performance.

## Introduction

A quarter of the market share of chemical intermediates and consumer products is produced by selective oxidation of hydrocarbon molecules. Taking into account the dimensions of these applications, selective formation of desired reaction products is of vital importance in terms of sustainable utilization of resources and carbon dioxide mitigation.^1^ Most frequently, complex mixed oxides are used as catalysts in heterogeneous processes applying molecular oxygen as an oxidant. The multiplicity of potential reaction pathways initiated by C–H activation on the surface of metal oxides,^2^ and the simultaneous occurrence of active oxygen species require catalysts that master selectivity predictively.

Thus research is focused on the identification of descriptors that provide directions in catalyst development and contribute to an improved understanding of relationships between reaction mechanisms and the dynamics of the atomistic surface structure.^3^,^4^ Facet sensitivity has been proposed as an important parameter that controls the establishment of selective single-site catalysts.^5^–^7^ The concept is of fundamental importance in heterogeneous catalysis and materials science.^8^–^12^ The effects of size, shape and termination of active catalyst particles has been most frequently discussed in metal-catalyzed reactions.^9^,^11^–^15^ Similarly, an impact of the catalyst particle morphology on catalytic properties has been found for nano-crystalline metal oxides.^14^,^16^–^18^ Shape-dependent activities in oxidation reactions over binary oxides such as MoO_3_,^19^,^20^ MgO,^21^–^23^ Co_3_O_4_,^24^,^25^ CeO_2_ 26–28 and Cu_2_O,^29^ and also complex oxides, such as single-phase MoVTeNb oxide,^6^,^30^ have been attributed to the specific activity of selected crystal planes.

A full understanding of activity–structure relationships over oxide catalysts is, however, challenging due to the complexity of metal oxide surfaces in terms of polarity,^31^ hydroxyl-hydrate cover,^32^ and defects.^33^–^35^ Furthermore, deviations from the bulk with respect to elemental composition and oxidation states have been reported.^36^,^37^ A thin surface layer detected on the surface of vanadium oxide-based catalysts accounts for a dynamic charge transfer between the bulk and surface, which is reflected in the response of the work function, electron affinity and surface potential barrier to the chemical potential of the gas phase in analogy to the behavior of gas sensors with strong impact on catalytic properties.^38^–^42^

In the present work we investigate the actual cause of structure sensitivity in oxidation catalysis over metal tungstate catalysts by studying the impact of shape and size of primary catalyst particles in the oxidative dehydrogenation (ODH) of propane as a relevant case study over a series of highly crystalline, but nano-structured manganese tungstate catalysts. Compared to previously reported activities of metal molybdates MMoO_4_ (M = Ni, Co, Mn, Mg and Zn),^43^–^47^ and tungstates MWO_4_ (M = Ni, Co, Zn, Fe and Ce)^48^,^49^ in the oxidative dehydrogenation of propane, nano-structured MnWO_4_ exhibits an exceptionally high specific reaction rate that has been attributed to active sites in a well-defined manganese oxide surface layer, which is formed under the specific conditions of hydrothermal synthesis.^50^,^51^ The result inspired us to prepare a series of phase-pure MnWO_4_ catalysts with different particle morphologies by adopting the hydrothermal synthesis conditions.

Rigorous analysis of bulk and surface structures is performed applying a variety of complementary techniques including multi-laser excitation Raman spectroscopy, X-ray photoelectron spectroscopy (XPS) and electron energy loss spectroscopy (EELS). *In situ* X-ray absorption spectroscopy (XAS) and operando Fourier transform infrared spectroscopy (FTIRS) are applied to gain insight into the sequence of reaction steps involved in selective and unselective oxidation.

Owing to the regular, well-defined surface structure of the nano-structured MnWO_4_ catalysts, direct spectroscopic evidence of propane activation and assistance of oxygen in the regeneration of active sites is provided. The findings unravel the origin of limited selectivity to valuable products in alkane oxidation over this type of catalyst and attach importance to the multifaceted appearance of the active site isolation concept, which has been postulated more than 50 years ago,^52^ transferring this knowledge to the age of big data.^53^

## Experimental and computational details

### Synthesis of catalysts

The hydrothermal synthesis of nano-structured MnWO_4_ was performed in an analytical autoclave HPM-PT-040-Mönch (Premex Reactor GmbH) adopting a method that has been reported previously.^54^ In the first step, a 0.2 M aqueous solution of Mn(NO_3_)_2_ (Mn(NO_3_)_2_·4H_2_O, 98%, Roth) was added to a 0.2 M aqueous solution of Na_2_WO_4_ (Na_2_WO_4_·2H_2_O, 99%, Sigma Aldrich) while stirring leading to a mixed solution of pH = 6.7. Subsequently, the pH of the mixed solution was adjusted to 6.3, 8.0, 9.1, and 9.9, respectively, by adding appropriate amounts of 0.1 M HNO_3_ (64–66%, Sigma Aldrich) or 0.1 M NaOH (98%, Alfa Aesar), respectively. The mixtures were transferred to the autoclave and the temperature was raised from 293 K to 453 K at a rate of 5 K min^–1^. The synthesis temperature was kept at 453 K for 12, 24, and 48 h, respectively. After cooling down the gel at a rate of 5 K min^–1^, the products of hydrothermal synthesis were isolated by centrifugation and washed twice with de-ionized water (MilliPore®). In the final step, the solids were dried in a muffle furnace in air at 353 K for 12 h. The products of hydrothermal synthesis were annealed in argon (flow rate: 50 mL min^–1^) at 673 K (heating rate: 5 K min^–1^) for 2 h using a rotary tube furnace (XERION ADVANCED HEATING Ofentechnik GmbH, Freiberg, Germany) resulting in seven phase-pure MnWO_4_ catalysts referred to as “freshly activated catalysts” in the following. The catalysts are called pH *x*.*x*@*y* h, where *x*.*x* corresponds to the pH value adjusted before hydrothermal synthesis and *y* corresponds to the hydrothermal synthesis time at 453 K.

### Catalyst characterization

High resolution TEM (HRTEM) and high resolution annular dark field scanning transmission electron microscopy (ADF-STEM) were performed on a double corrected JEOL JEM-ARM200F equipped with CEOS CESCOR, and CEOS CETCOR hexapole aberration correctors for probe and image forming lenses, respectively, and a cold field emission gun (CFEG). The acceleration voltage was set to 200 kV. The specimens were prepared by drop deposition from an ethanolic suspension onto lacey-carbon-coated Cu grids. Field emission scanning electron microscopy (FESEM) was carried out with a Hitachi S4800 instrument operating at 5 kV. STEM-EELS measurements were conducted with a Gatan Quantum Image Filter. The spectra were dark current and background corrected.

The surface area of the catalysts was determined using a volumetric N_2_ physisorption apparatus (Autosorb-6-B, Quantachrome). The catalysts were treated in a dynamic vacuum at 573 K for 2 h prior to adsorption of nitrogen at 77 K. The specific surface area was calculated according to the BET method in the pressure range *p*/*p*_0_ = 0.05–0.35 using 11 data points.

X-ray diffraction (XRD) measurements were performed in Bragg–Brentano geometry on a Bruker AXS D8 Advance II theta/theta diffractometer, using Ni filtered Cu Kα radiation and a position sensitive LynxEye silicon strip detector. The XRD data were evaluated by whole powder pattern fitting according to the Rietveld method as implemented in the TOPAS software [version 4.2, copyright 1999–2009 Bruker AXS] taking into account the anisotropic crystallite shape of the catalyst particles as described in detail before.^51^

Raman measurements have been carried out using a confocal Raman spectroscopy system (S&I Spectroscopy & Imaging GmbH) including a monochromator (Princeton Instruments) connected with a liquid nitrogen cooled CCD camera. The Raman spectra were obtained using a variety of individual lasers operating at 532 nm, 488 nm, 457 nm, 442 nm and 355 nm excitation, respectively. A laser power lower than 0.50 mW was used to avoid beam damage. In addition, a He–Ne 632.8 nm laser (1.5 mW) was applied for excitation using a Horiba Jobin LABRAM instrument equipped with a microscope (Olympus). A pressed wafer of the catalyst was mounted on the sample holder in air at ambient temperature without pretreatment when recording the spectrum.

Operando FTIRS measurements were carried out using a Varian 670 FTIR spectrometer equipped with a MCT detector. The spectra were recorded at a resolution of 2 cm^–1^ accumulating 128 scans. Self-supported wafers (area weight of 28–45 mg cm^–2^) were transferred into an IR cell that is connected to a vacuum line and a gas delivery system. Prior to the reaction, the catalysts were treated at 673 K in a flow of 50 mL min^–1^ He for 2 hours. The oxidative dehydrogenation of propane was investigated at atmospheric pressure and 673 K using C_3_H_8_/O_2_/He = 10/5/85 feed at a flow rate of 10 and 5 mL min^–1^, respectively. The product was analyzed using an on-line gas chromatograph (Varian Mirco GC 490) equipped with 10 m Pora Plot Q and 1 m COX column modules and with TCD detectors.

*In situ* diffuse reflectance infrared Fourier transform spectroscopy (DRIFTS) was conducted using an Agilent Cary 680 FTIR spectrometer equipped with a MCT detector at a spectral resolution of 4 cm^–1^ and accumulation of 512 scans. An *in situ* cell (Harrick Praying MantisTM diffuse reflectance attachment DRP-P72 in combination with a low temperature CHC-CHA reaction chamber with ZnSe windows) was used. Spectra were taken at 77 K or 313 K, respectively, after appropriate pretreatment in the reaction chamber. The amount of the catalyst used was approximately 50 mg. 50 mL min^–1^ argon was purged during heating, and a heating rate of 5 K min^–1^ was applied.

X-ray photoelectron spectra were recorded at room temperature, using non-monochromatized Al Kα (1486.6 eV) excitation and a hemispherical analyzer (Phoibos 150, SPECS). The binding energy scale was calibrated by the standard Au 4f_7/2_ and Cu 2p_3/2_ procedure. Theoretical cross sections were used to calculate the elemental composition.^55^

Near edge X-ray absorption fine structure (NEXAFS) analysis was conducted at the near ambient pressure XPS end station of the ISISS beam line at HZB/BESSY II (Berlin, Germany). Details of the setup have been published earlier.^56^ The experiments were performed at a total pressure of 0.25 mbar in O_2_/He or C_3_H_8_/O_2_/He mixtures with a total gas flow of 1.2 sccm at temperatures between 573 K and 673 K. NEXAFS spectra were recorded in total electron yield (TEY) mode. Due to the low inelastic mean free path of electrons in solids, electron yield X-ray absorption spectroscopy is more surface sensitive than fluorescence based techniques.

Temperature-programmed oxidation (TPO) was performed in a fixed-bed quartz reactor using 300 mg of the catalyst. Prior to the first TPO measurement, the catalyst was pretreated at 673 K for 2 h in Ar (flow rate: 50 mL min^–1^, heating rate: 5 K min^–1^). The TPO measurement was carried out in a mixture 0.24% O_2_/He (flow rate 100 mL min^–1^), applying a heating rate of 5 K min^–1^ and a holding time at 673 K of 60 min. O_2_ consumption was monitored with a paramagnetic detector.

Oxidative dehydrogenation of propane was carried out using a setup for partial oxidation (Integrated Lab Solutions) with 8 fixed bed quartz reactors (6 mm inner diameter) in parallel. Each reactor was equipped with a thermocouple for measuring the temperature inside the catalyst bed containing 100–300 mg of catalyst previously pressed and sieved to a particle size of 250–355 μm. The catalytic performances were determined at atmospheric pressure. The reactant feed was composed of C_3_H_8_, O_2_, and N_2_ in a volume ratio of 10 : 5 : 85 at a total flow rate of 10–20 mL min^–1^. The gas mixtures were analyzed by using an online gas chromatograph (Agilent 7890). A system of Plot-Q and Plot-MoleSieve 5A columns connected to a thermal conductivity detector (TCD) separated the permanent gases CO, CO_2_, N_2_, O_2_, and CH_4._ A system of a FFAP and a Plot-Q column connected to a flame ionization detector (FID) allowed the separation of C2–C3 hydrocarbons and oxygenates. The freshly activated catalysts were loaded into the reactor and the temperature was increased in the feed to reaction temperature 673 K. Conversion of propane *X*, selectivity to product j *S*_j_ and propane consumption rates *r* were calculated according to eqn (1)–(3)1
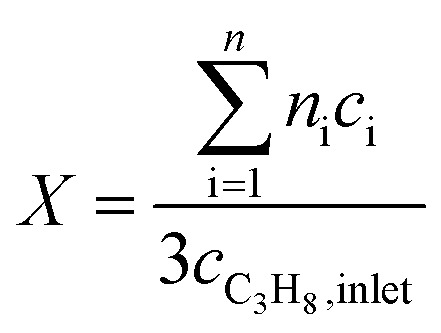

2
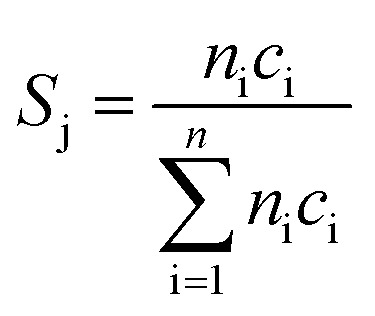

3
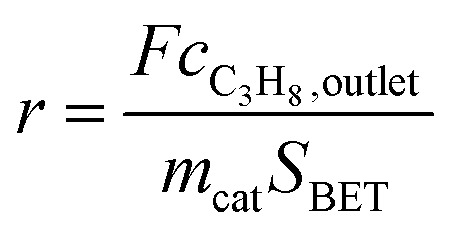
where *n*_i_ is the carbon atom number in the product molecule i, *c*_i_ is the concentration of the product i analyzed by gas chromatography, *c*_C_3_H_8_, inlet_ and *c*_C_3_H_8_, outlet_ are the concentrations of propane in the feed gas at the reactor inlet and outlet, *F* is the total flow rate of the inlet gas stream, *m*_cat_ is the weight of the catalyst loaded into the reactor, and *S*_BET_ is the specific surface area of the catalyst.

### DFT calculations

All DFT calculations were performed using the Quantum ESPRESSO package^57^ using ultrasoft pseudopotentials (PP) from the Original QE PP library and the exchange and correlation potential developed by Perdew, Burke, and Ernzerhof^58^ with Cococcioni's and de Gironcoli's simplified rotationally invariant Hubbard U applied to the Mn d-orbitals.^59^ Wave functions were expanded in a planewave basis set with a kinetic energy (charge density) cutoff of 30 Ry (300 Ry). A *k*-point mesh equivalent to (6 × 6 × 1) for the (010) surface unit cell was used. The Mn-rich MnWO_4_ surfaces were modeled with a symmetric 54 atom slab (4 layers of stoichiometric MnWO_4_ and an additional layer of MnO_4_). The initial ion positions and lattice parameters for MnWO_4_ under ambient conditions were taken from the crystal structure analysis.^60^ The ion positions of the surface atoms were allowed to relax while holding the central 24 atoms (2 layer of stoichiometric MnWO_4_) and the lattice parameters fixed. Approximately 20 Å of vacuum was used to separate periodic images. The Mn atoms were ordered ferromagnetically in the (010) plane and antiferromagnetically in alternating planes in the direction normal to the surface.

The value of *U* (3.05 eV) applied to the Mn d-orbitals in this work was computed for Mn in a (2 × 2 × 2) type I antiferromagnetic supercell of MnO using linear response.^59^ The calculation was performed using ion positions and lattice parameters for MnO taken from the crystallography open database, and an (8 × 8 × 8) *k*-point mesh was employed with Marzari–Vanderbilt cold smearing with a smearing parameter of 0.001 Ry.^61^ Subsequent optimization of the experimental lattice parameters with *U* = 3.05 eV led to a minor expansion of the lattice, from 4.45 Å to 4.53 Å.

The Mn rich surface model employed in this work was developed in accordance with previous TEM findings, where the (010) surface was found to be terminated in an Mn–O–Mn motif.^50^ Both sides of the slab were then hydroxylated and the ion positions were allowed to relax to their ground states before computing the OH stretching frequencies by using finite displacements of the H and O atoms involved in OH bonding. Note that using only finite displacements of H resulted in changes of less than 1 cm^–1^ in the computed vibrational modes.

## Results and discussion

Polycrystalline MnWO_4_ catalysts were prepared by hydrothermal synthesis followed by thermal treatment in argon at 673 K. Particle shape and size were adjusted through variation of pH^51^ and synthesis time. The synthesis parameters and general characteristics of the different catalysts are summarized in Table 1. The phase-purity of the catalysts was confirmed by Rietveld refinement (ESI, Fig. S1†). All catalysts are solely composed of the wolframite-type structure, which is based on a distorted hexagonal closed packing of oxygen atoms with Mn and W atoms each occupying one fourth of the octahedral interstices.^62^ The *ortho*-tungstate family M^II^(W,Mo)^VI^O_4_ comprises molybdates and tungstates of bivalent metals with an ionic radius smaller than 0.77 Å (M^II^ = Fe, Mn, Co, Ni, Mg, Zn).^54^ Distorted WO_6_ octahedra form zigzag chains by sharing edges along the [001] crystallographic axis. Similarly, MnO_6_ octahedra form zigzag chains along the same crystallographic axis by sharing corners with WO_6_ octahedra. Along the [100] axis the two types of zigzag chains stack alternately by edge-sharing.^50^,^51^

**Table 1:** General information about the catalysts prepared by hydrothermal synthesis at different pH and times and activated in Ar at 673 K for 2 hours

Catalyst	Catalyst ID^*a*^	pH^*b*^	Synthesis time^*c*^ (hours)	Aspect ratio^*d*^ (AR)	*S*_BET_ (m^2^ g^–1^)
pH 6.3@12 h	19 112	6.3	12	1.5	25.9
pH 6.7@12 h	19 113	6.7	12	1.7	22.9
pH 8.0@12 h	19 114	8.0	12	3.2	22.1
pH 9.1@12 h	19 251	9.1	12	3.5	21.0
pH 9.9@12 h	19 116	9.9	12	5.1	28.7
pH 9.9@24 h	20 111	9.9	24	6.8	21.5
pH 9.9@48 h	20 112	9.9	48	5.4	19.2

^*a*^Required to identify clearly different batches of catalyst synthesis.

^*b*^pH value adjusted before hydrothermal synthesis.

^*c*^Time of hydrothermal synthesis at 453 K.

^*d*^Aspect ratio of primary catalyst particles determined by electron microscopy.

**Fig. 1:**
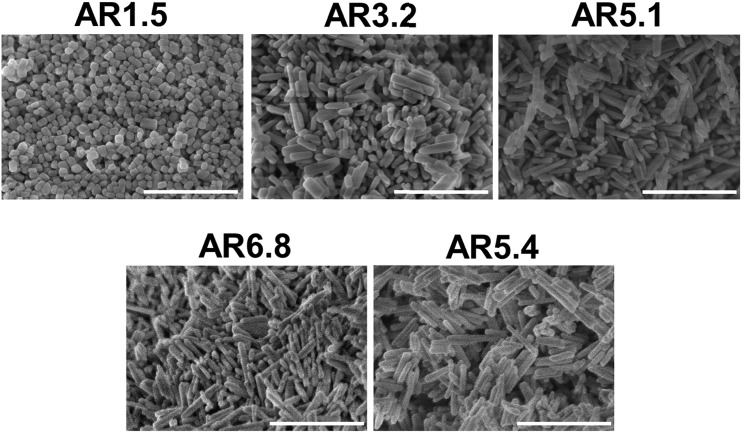
SEM images of the as-synthesized catalysts pH 6.3@12 h (AR = 1.5), pH 8.0@12 h (AR = 3.2), pH 9.9@12 h (AR = 5.1), pH 9.9@24 h (AR = 6.8), and pH 9.9@48 h (AR = 5.4). The scale bar is 500 nm.

SEM images (Fig. 1) of the as-synthesized MnWO_4_ nano-oxides indicate morphological alteration, which is induced by changes of the synthesis protocol (pH and time). The variations can be expressed by different aspect ratios (AR) of the primary catalyst particles (Table 1). Fig. 1 shows exemplarily catalysts with very different aspect ratios. Cube-like particles are obtained at low pH (6.3 and 6.7), while with increasing pH (8.0–9.9) and dwell time (12–24 h) the AR increases, which is reflected in a pronounced rod-like morphology. Only for higher dwell times (48 h) at the highest pH (9.9) the AR decreases again, which is attributed to dissolution of the nano-rods in the highly alkaline medium over prolonged time.

**Fig. 2:**
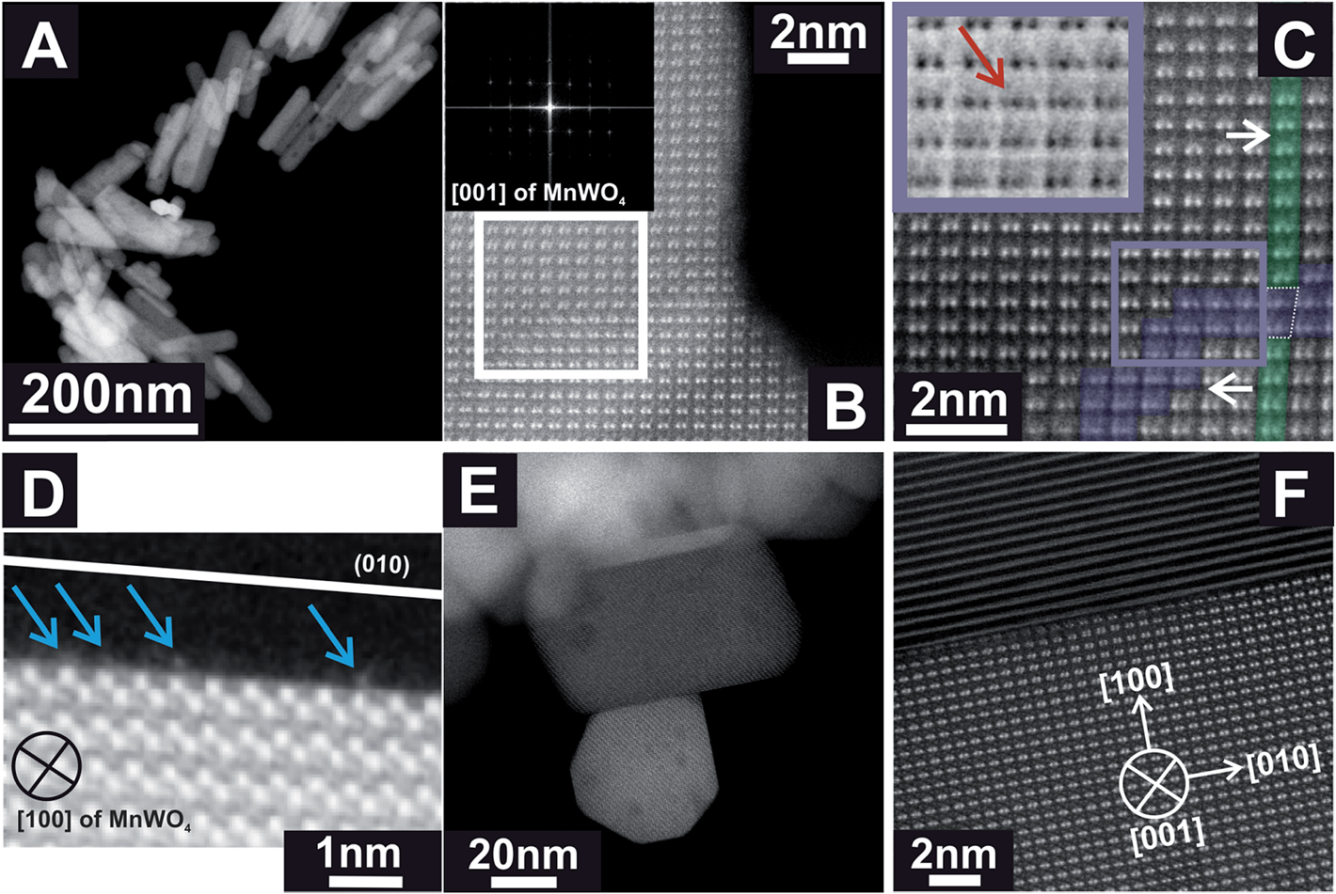
ADF-STEM images of MnWO_4_ nanoparticles pH 9.9@12 h (AR = 5.1) (A–D) and pH 6.3@12 h (AR = 1.5) (E and F) at different magnifications. The inset in (B) denotes the Fast Fourier Transform (FFT) of the presented area. (C) shows a magnified FFT filtered image of the region highlighted by the white box in (B). The white arrows in (C) correspond to the shift direction. The violet color in (C) displays a region of a W trimeric intergrowth, while the green areas are attributed to W dimers before and after the intergrowth. The inset in (C) represents a magnified inverted ADF-STEM image of the region highlighted by the violet box in (C). The red arrow points to a W trimer. (D) demonstrates a (010) terminated surface of a MnWO_4_ crystal viewed along [100]. The blue arrows highlight surface Mn sites.

Previous STEM images have shown a defective character of rods with an aspect ratio of 5.1, which can be described as extended bulk defects that propagate to the surface.^50^ The particle length determined by TEM differed from the particle dimension along the The particle length determined by TEM differed from the particle dimension along the 〈001〉 direction determined by XRD.001 The particle length determined by TEM differed from the particle dimension along the 〈001〉 direction determined by XRD. direction determined by XRD.^51^ Hence, it was concluded that the rod-like particles are composed of a number of crystals along the Hence, it was concluded that the rod-like particles are composed of a number of crystals along the 〈001〉 direction. In-depth analysis of annular dark-field scanning transmission electron microscopy (ADF-STEM) images of the catalyst pH 9.9@12 h with an aspect ratio of 5.1 (001 Hence, it was concluded that the rod-like particles are composed of a number of crystals along the 〈001〉 direction. In-depth analysis of annular dark-field scanning transmission electron microscopy (ADF-STEM) images of the catalyst pH 9.9@12 h with an aspect ratio of 5.1 ( direction. In-depth analysis of annular dark-field scanning transmission electron microscopy (ADF-STEM) images of the catalyst pH 9.9@12 h with an aspect ratio of 5.1 (Fig. 2A–D) reveals that the extended defects represent ) reveals that the extended defects represent 〈WW_3_O_*y*_〉 trimers formed by condensation of two MnWO trimers formed by condensation of two MnWO_4_ crystals. The defects propagate to the [010] surface. The crystals before and after the trimeric intergrowth are shifted by one W atom along [010] (Fig. 2C). The high-resolution ADF-STEM image in Fig. 2D displays a surface terminated with the (010) plane of MnWO_4_ viewed along [100]. The surface exhibits a partial enrichment of manganese (Mn atoms highlighted by the blue arrows). In contrast, ADF-STEM images of MnWO_4_ pH 6.3@12 h with a low aspect ratio (1.5) (Fig. 2E and F) reveal the absence of extended defects. For example, the high resolution ADF-STEM micrograph presented in Fig. 2F reproduces the boundary region of two condensed MnWO_4_ crystals, which do not themselves show irregular geometric structures.

**Fig. 3:**
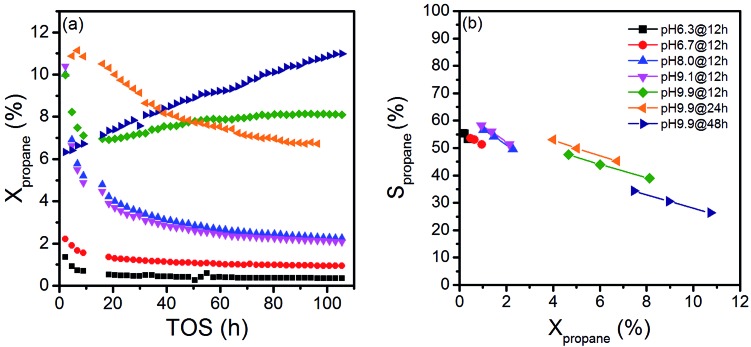
(a) Propane conversions as a function of time-on-stream (TOS) (*T* = 673 K, *W*/*F* = 1.8 g s mL^–1^, feed: 10% C_3_H_8_, 5% O_2_ and 85% N_2_), and (b) selectivity as a function of conversion under steady state conditions (*t* > 100 h, *T* = 673 K, *W*/*F* = 0.9–2.4 g s mL^–1^, feed: 10% C_3_H_8_, 5% O_2_ and 85% N_2_).

The variation in synthesis conditions is not only reflected in different particle morphologies, but also in varying activity and selectivity in propane oxidation. The catalysts with a low aspect ratio prepared at pH < 9.9 that lack defects show lower catalytic activity compared to the catalysts prepared at pH = 9.9 that are characterized by a higher abundance of bulk and surface defects (Fig. 3). Propane and oxygen conversions were kept below 12% and 30% (oxygen conversion not shown), respectively, to avoid mass transport limitation and significant hotspots. Furthermore, the maximum reaction temperature was limited to 673 K to minimize contributions of homogenous gas-phase reactions. All catalysts produce exclusively propene, CO and CO_2_.

The activity changes with time on stream (Fig. 3a). All catalysts prepared after adjustment of a starting pH < 9.9 deactivate gradually. No systematic behavior is observed for the three catalysts synthesized applying different hydrothermal times at pH = 9.9. The catalyst pH 9.9@12 h initially deactivates, but at *t* > 20 h the activity increases again with time on stream. In contrast, the activity of the catalyst pH 9.9@24 h passes through a maximum at *t* = 10 h, while the catalyst prepared for 48 h pH 9.9@48 h exhibits increasing activity with time on stream without reaching the steady state. For the sake of convenience, the state after 100 h time on stream is called steady-state also for this catalyst. These observations indicate that all catalysts undergo significant modifications under reaction conditions.

**Fig. 4:**
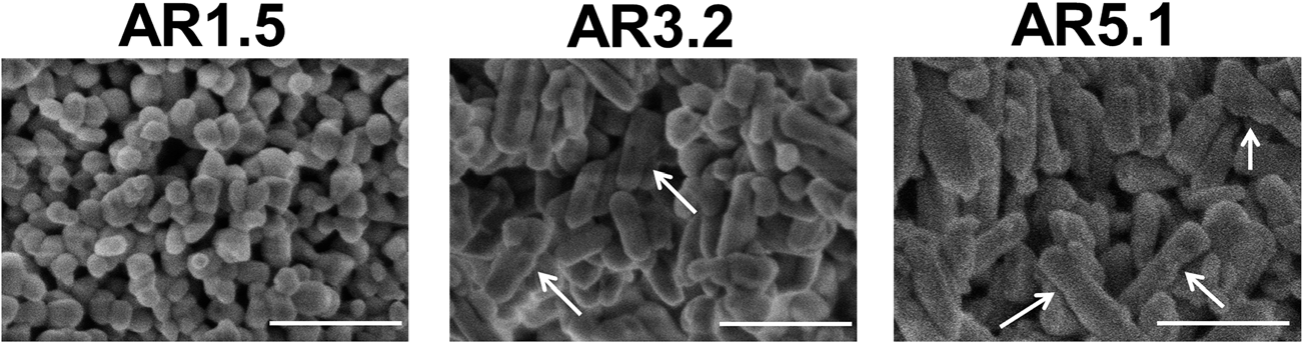
SEM images of the catalysts pH 6.3@12 h (AR = 1.5), pH 8.0@12 h (AR = 3.2), and pH 9.9@12 h (AR = 5.1) after oxidative dehydrogenation of propane at *T* = 673 K. The arrows denote reconstructed surfaces. The scale bar is 200 nm.

**Fig. 5:**
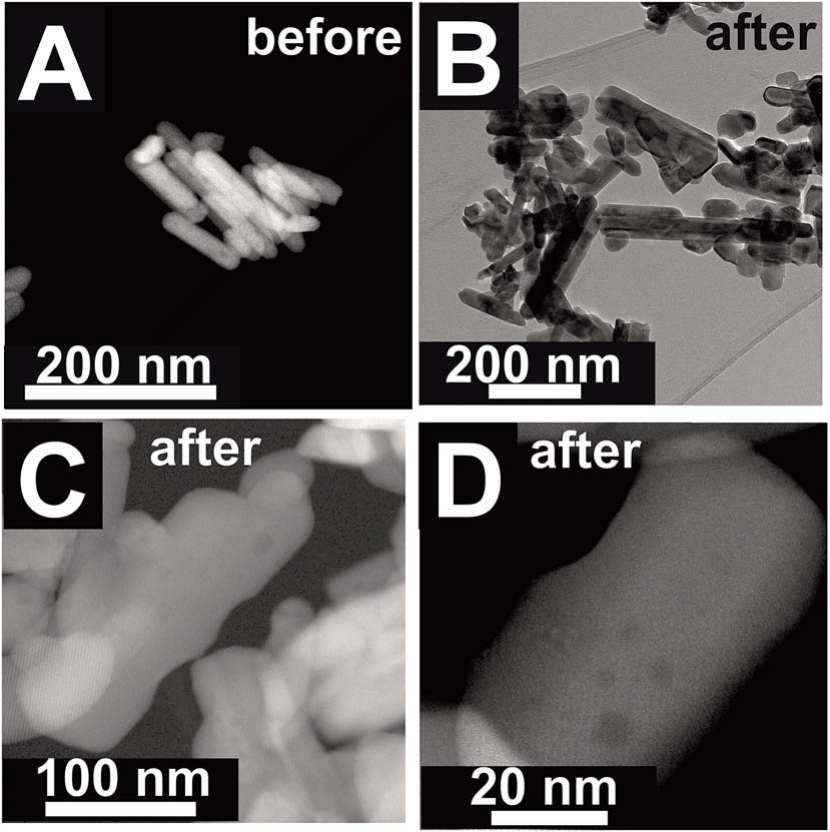
(S)TEM images of pH 9.9@12 h (AR = 5.1) before (A) and after (B–D) oxidative dehydrogenation of propane at *T* = 673 K at different magnifications.

The changes are neither caused by destruction of the bulk crystal structure as shown by XRD (Fig. S2†), nor by significant changes in the overall morphology (Fig. 4). SEM (Fig. 4, AR = 3.2 and AR = 5.1) and (S)TEM (Fig. 5) images of the spent rod-like MnWO_4_ with a higher aspect ratio display a partially reconstructed surface (see arrows in Fig. 4) along the growth direction, which is not observed in the SEM images of the used cube-like MnWO_4_ (Fig. 4, AR = 1.5). High-resolution ADF-STEM images in Fig. 5C and D suggest that the surface reconstruction is independent of the crystallographic surface termination. Compared to the fresh catalyst (Fig. 2A and 5A), a higher fraction of low aspect ratio particles is observed in the overview TEM image of the spent catalyst pH 9.9@12 h (Fig. 5B), most likely caused by splitting of the rods into smaller fractions under reaction conditions. This is also supported by anisotropic Rietveld refinement of the XRD patterns of the used catalysts (Table S1†). The analysis suggests that in particular the longer rods are composed of several coherently scattering domains stacked in the crystallographic *c* direction, and the number of these domains increases after the use of the catalyst (Table S1†). In contrast, MnWO_4_ nano-cubes (pH 6.3@12 h (AR = 1.5)) (Fig. 1 and 4) are modified structurally very little by the reaction atmosphere, which is in agreement with the moderate deactivation of the catalyst (Fig. 3a, black data points).

Due to the marked changes in the activity from the initial to the steady-state, the results of spectroscopic surface characterization of freshly activated catalysts are only used for the interpretation of initial catalytic performance measured at *t* = 0. Differences in the initial propane conversion over the catalyst series (Fig. 3a) cannot be related to differences in the specific surface area, because the specific surface area of all catalysts is similar and varies between 20 and 30 m^2^ g^–1^ (Table 1). The consumption rates of propane normalized to the specific surface area and measured at *t* = 0 are presented in Table 2. The table also comprises specific reaction rates measured under steady-state conditions (*t* > 100 h) for comparison. Systematic correlations between the aspect ratio and the normalized catalytic activity were not observed, neither at *t* = 0 h nor at *t* > 100 h. The catalysts prepared at the highest pH are generally more active compared to the catalysts prepared at lower pH (Fig. 3, Table 2), but no clear trend becomes obvious.

**Table 2:** Normalized consumption rates of propane at *t* = 0 and under steady-state conditions (*t* > 100 h, *T* = 673 K, *W*/*F* = 1.8 g s mL^–1^, feed: 10% C_3_H_8_, 5% O_2_ and 85% N_2_) in the oxidative dehydrogenation of propane over nano-structured MnWO_4_ catalysts, oxygen defect densities on the freshly activated catalysts determined by TPO, and elemental composition near the surface of fresh and used catalysts determined by XPS

Catalyst	Aspect ratio^*a*^ (AR)	*r*_propane, initial (*t*=0)_ (10^–9^ mol m^–2^ s^–1^)	Mn^*b*^/W	Na^*b*^/(Mn + W)	Oxygen defect density^*c*^ 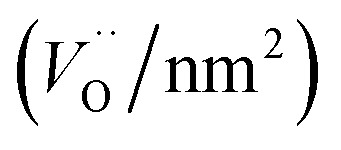	*r*_propane, steady-state_ (10^–9^ mol m^–2^ s^–1^)	Mn^*d*^/W
pH 6.3@12 h	1.5	1.1	1.13	0.11	0.57	0.36	1.07
pH 6.7@12 h	1.7	2.4	1.20	0.13	0.75	1.1	1.13
pH 8.0@12 h	3.2	10.0	1.30	0.10	3.81	2.9	1.25
pH 9.1@12 h	3.5	9.1	1.32	0.079	4	2.8	1.23
pH 9.9@12 h	5.1	9.4	1.30	0.10	3.06	9.2	1.26
pH 9.9@24 h	6.8	11.4	1.32	0.17	2.87	7.1	n.d.
pH 9.9@48 h	5.4	7.6	1.27	0.056	0.67	11.3	n.d.

^*a*^Determined by electron microscopy.

^*b*^Determined by X-ray photoelectron spectroscopy (XPS) of the fresh thermally activated catalysts.

^*c*^Determined by temperature-programmed oxidation (TPO) of the fresh thermally activated catalyst.

^*d*^Determined by X-ray photoelectron spectroscopy (XPS) of catalysts used in oxidative dehydrogenation of propane.

The selectivity as a function of conversion under steady-state conditions is presented in Fig. 3b. The selectivity of propene decreases with increasing propane conversion in agreement with consecutive reactions of formed propene resulting finally in the formation of CO and CO_2_. The *S*–*X* trajectories seem to follow roughly the same trend for all seven catalysts suggesting no significant changes in the reaction network within the series of catalysts. Extrapolation to zero percent conversion suggests that parallel reactions of propane leading to deep oxidation occur to a certain extent. Differences in selectivity at comparable conversion comprise only up to 10% among all catalysts. In the following discussion, we will therefore concentrate on an explanation of differences in activity.

**Fig. 6:**
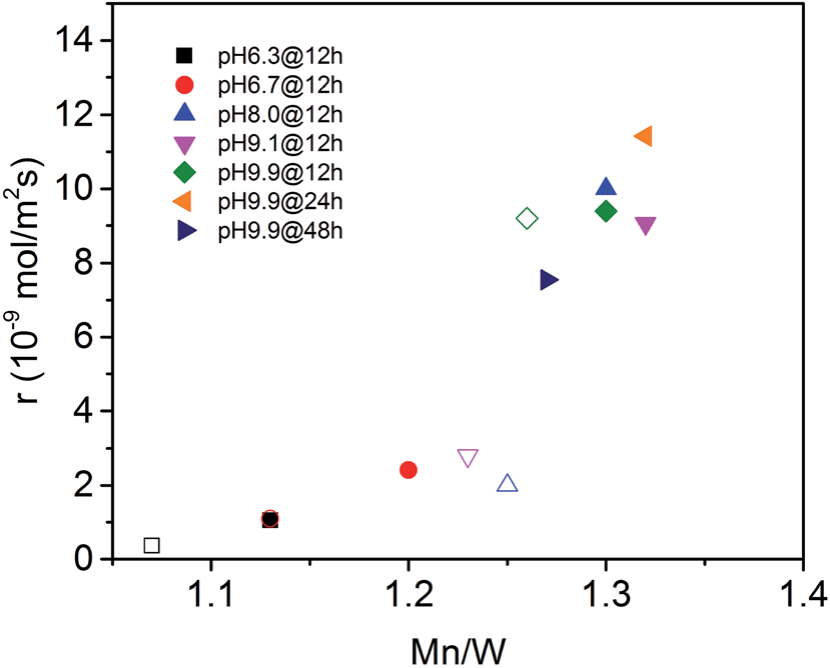
Initial consumption rate of propane (filled symbols) and consumption rate of propane in the steady-state (open symbols) as a function of the Mn to W atomic ratio in the near surface region determined by XPS on the fresh (filled symbols) and used (open symbols) catalysts (see also Table 2).

The surface properties of the catalysts have been studied by spectroscopic techniques and temperature-programmed oxidation. The molar Mn/W ratio determined by photoelectron spectroscopy for all seven catalysts is presented in Table 2 and Fig. 6. The increase in activity at *t* = 0 with increasing pH during hydrothermal synthesis can be attributed to an enrichment of Mn in the near surface region (Fig. 6) in agreement with facet-specific dissolution–recrystallization processes under hydrothermal conditions that are responsible for the development of the rod-like shape of the primary catalyst particles. The mechanism of particle growth has been discussed in our previous publication.^51^ In short, the basic conditions of the hydrothermal synthesis provoke preferential leaching of tungsten oxide moieties at (010) crystal planes. Re-crystallization proceeds *via* condensation of the dissolved [WO_4_]^2–^ species with W–O(H) groups at high-energy (001) basal planes resulting in an anisotropic growth along the crystallographic species with W–O(H) groups at high-energy (001) basal planes resulting in an anisotropic growth along the crystallographic 〈001〉 direction and an enrichment of Mn(OH)001 species with W–O(H) groups at high-energy (001) basal planes resulting in an anisotropic growth along the crystallographic 〈001〉 direction and an enrichment of Mn(OH) direction and an enrichment of Mn(OH)_*x*_ species on the surface. The Mn/W ratio in the bulk is in agreement with the stoichiometry of the compound. The Mn/W ratio near the surface (Table 2) increases with increasing starting pH, and remains almost constant with increasing hydrothermal synthesis time at pH = 9.9 except for the catalyst pH 9.9@48 h. For the latter the Mn surface concentration decreases again slightly perhaps due to re-dissolution or increasing aggregation of manganese oxide clusters due to the extended hydrothermal synthesis time under basic conditions.

The catalytic activity of the nano-structured MnWO_4_ catalysts is clearly associated with the surface concentration of manganese as evidenced by the trend in the graphical representation of the reaction rate at *t* = 0 as a function of the Mn/W atomic ratio determined by XPS near the surface of the freshly activated catalysts (filled symbols) (Fig. 6). The trend is confirmed by taking into account the rates measured under steady-state conditions and the Mn/W ratios of the used catalysts (open symbols). The correlation is not linear, which indicates that clustering effects may play a role with increasing coverage of the surface by manganese oxide species. Based on the trend, the rate appears to be zero at a Mn/W ratio of 1, which is in agreement with the very low activity of the commercial MnWO_4_ reference sample that was not prepared by hydrothermal synthesis and not exposed to surface leaching.^50^

Although the solids were carefully washed after hydrothermal synthesis, all catalysts contain sodium impurities derived from Na_2_WO_4_ used as the tungsten precursor or NaOH applied to adjust the pH (Table 2). Sodium may act as catalyst poison; however, no systematic trend in terms of sodium content, synthesis conditions, activity and selectivity (Table 2, Fig. 3) is noted.

**Fig. 7:**
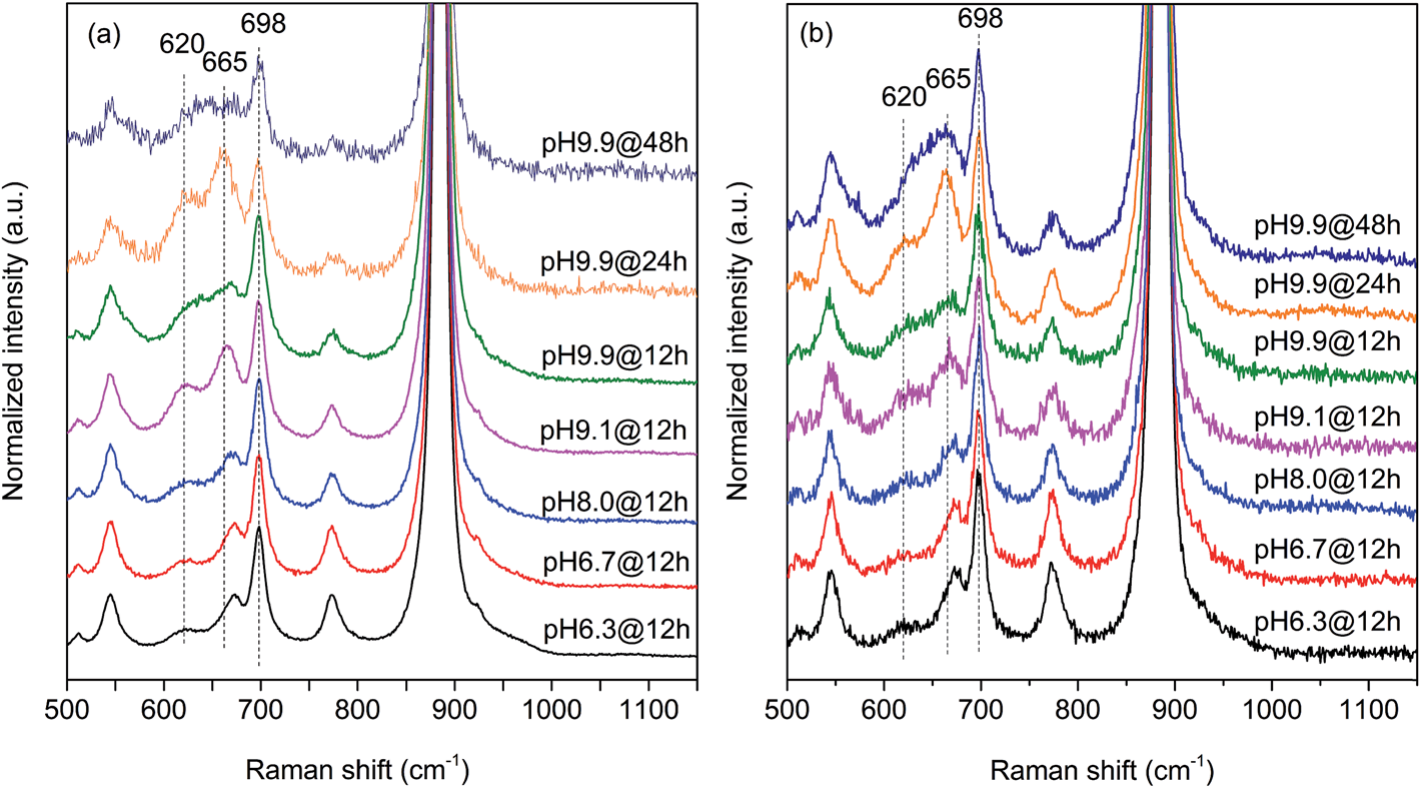
Raman spectra recorded using excitation at 532 nm of (a) freshly activated nano-structured MnWO_4_ catalysts and (b) catalysts after oxidative dehydrogenation of propane. The spectra were normalized by the maximum intensity followed by background subtraction.

**Fig. 8:**
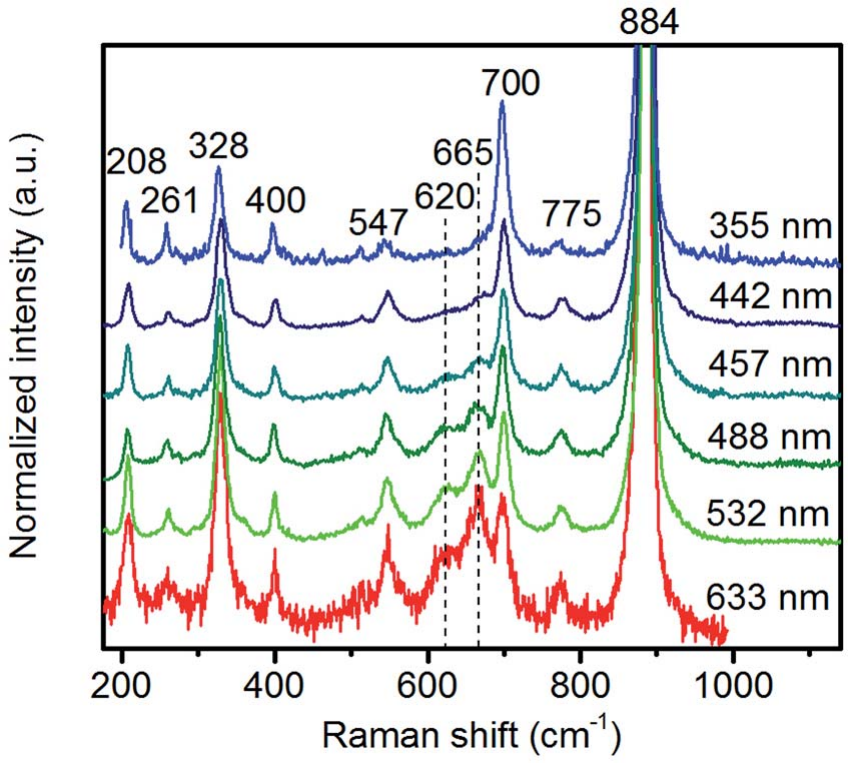
Raman spectra of pH 9.9@12 h recorded using different excitation energies. The applied laser wavelength is denoted on the right side of the spectra.

In agreement with the increased Mn/W ratio on the surface of the freshly activated catalysts measured by XPS, surface manganese oxide species that do not belong to the bulk crystal structure of MnWO_4_ have been detected by Raman spectroscopy (Fig. 7 and 8). Fig. 7 presents the Raman spectra of all seven catalysts recorded using an excitation wavelength of 532 nm. The spectroscopic patterns are consistent with those of single-crystalline manganese tungstate reported in the literature,^63^,^64^ except for two additional peaks centered at 620 and 665 cm^–1^. These two bands were attributed to surface MnO_*x*_ species.^50^,^65^ Because the translational symmetry is broken at the surface, observation of vibrational modes originating from surface Mn–O(H)–Mn species is possible by Raman spectroscopy, although this method is normally a bulk analysis technique. The vibrational frequency of 665 cm^–1^ actually coincides with the A_1g_ mode of MnO_6_ units in the Mn_3_O_4_ spinel structure that contains manganese in a mixed +II/+III oxidation state.^66^

Multi-laser excitation Raman spectroscopic analysis of the pH 9.9@12 h catalyst (Fig. 8) reveals a profound influence of the excitation energy on the relative intensity of the Raman bands at 620 and 665 cm^–1^ pointing to a resonance Raman enhancement effect,^67^ sensitive to surface Mn–O(H)–Mn species that are characterized by electronic transitions at lower energy typical of Mn–O charge transfer bands.^68^,^69^ Moreover, this observation demonstrates that the corresponding surface Mn oxide species are structurally separated from the bulk. Manganese may be anchored on the outermost layer by forming bridging Mn–O–W and Mn–O–Mn bonds as well as hydroxyl groups. The bands are clearly visible in the spectrum measured applying an excitation wavelength of 532 nm. Their intensities with respect to the fundamental modes of MnWO_4_ increase further by using the 633 nm laser; however, the signal to noise ratio of the latter spectrum is rather low. Therefore, the comparison of all catalysts, which is presented in Fig. 7, was performed using an excitation wavelength of 532 nm.

The two bands at 620 and 665 cm^–1^ are still detectable in the Raman spectra of the used catalysts (Fig. 7b), but their relative intensities *I*_620_/*I*_885_ and *I*_665_/*I*_885_ with respect to the A_g_(8) mode of bulk MnWO_4_ at 885 cm^–1^ (an O_2_–W stretching vibration) are decreased for catalysts pH 9.9@12 h and pH 9.9@24 h, which is in accordance with the diminished steady-state activity of these catalysts (Fig. 3a). In contrast, the relative intensities of the two bands are increased in the case of used pH 9.9@48 h in agreement with the activity that increases with time on stream. However, no direct correlation between these intensity ratios and catalytic performance was observed suggesting that the corresponding manganese oxide species are just precursors of active sites, but not quantitatively relevant in catalysis.

**Fig. 9:**
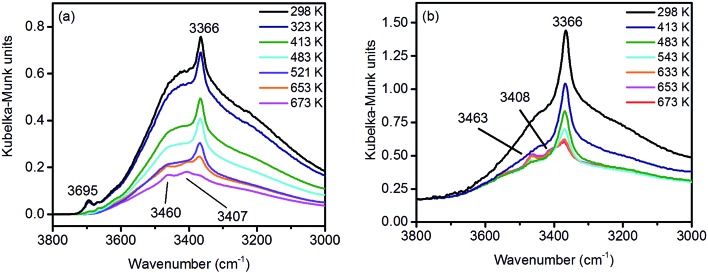
*In situ* DRIFT spectra of (a) pH 6.3@12 h and (b) pH 9.9@12 h catalysts during thermal pre-treatment in argon.

**Fig. 10:**
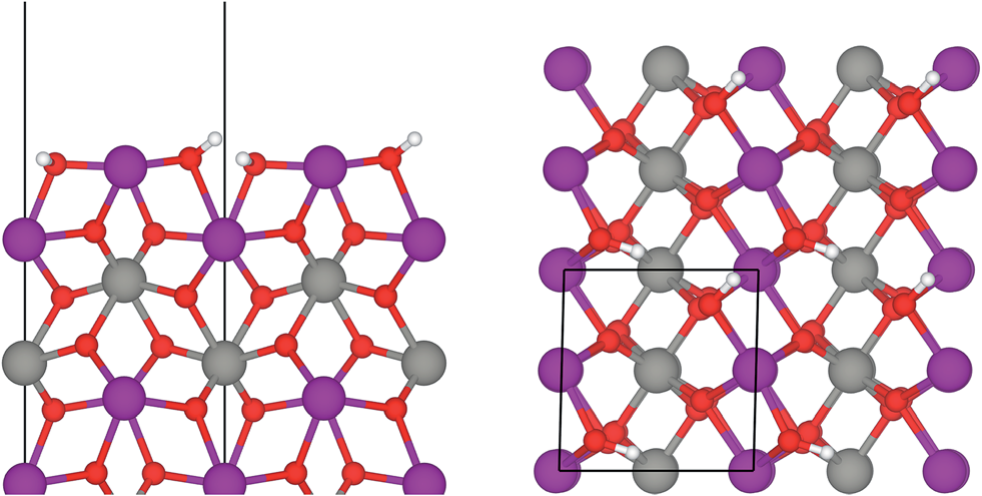
Ball-and-stick model of the Mn-rich termination of a MnWO_4_ (010) surface viewed along the (010) surface viewed along the 〈100〉 axis (left) and the 〈010〉 axis (right). Purple, grey, red and white balls represent Mn, W, O and H atoms, respectively. The solid black line shows the surface unit cell.100 (010) surface viewed along the 〈100〉 axis (left) and the 〈010〉 axis (right). Purple, grey, red and white balls represent Mn, W, O and H atoms, respectively. The solid black line shows the surface unit cell. axis (left) and the (010) surface viewed along the 〈100〉 axis (left) and the 〈010〉 axis (right). Purple, grey, red and white balls represent Mn, W, O and H atoms, respectively. The solid black line shows the surface unit cell.010 (010) surface viewed along the 〈100〉 axis (left) and the 〈010〉 axis (right). Purple, grey, red and white balls represent Mn, W, O and H atoms, respectively. The solid black line shows the surface unit cell. axis (right). Purple, grey, red and white balls represent Mn, W, O and H atoms, respectively. The solid black line shows the surface unit cell.

Under ambient conditions the catalysts are covered by a hydrate-hydroxyl shell as evidenced by DRIFTS of the catalysts pH 6.3@12 h and pH 9.9@12 h presented as examples for low and high aspect ratios, respectively, during catalyst pretreatment in Fig. 9. The broad feature in the range between 3700 and 3000 cm^–1^ corresponds to physisorbed water, which cannot be removed completely even after treatment in Ar at 673 K. A sharp band at 3695 cm^–1^ due to isolated W–OH groups^70^ observed only in the spectra of the catalyst pH 6.3@12 h synthesized at lower pH (Fig. 9a) disappears at temperatures between 373 K and 473 K due to dehydroxylation. Weak features at around 3463 and 3408 cm^–1^ and an intense band at 3366 cm^–1^ are visible in the spectra of all catalysts after partial dehydroxylation at 673 K. The intensity of the most intense peak at 3366 cm^–1^ normalized to the area weight of the respective pellet shows a trend (Fig. S3†) similar to that of the Mn/W ratio determined by XPS (Fig. 6) strongly supporting the assignment of the band to hydroxyl groups that coordinate to outermost Mn ions.^51^ Formation of a well-ordered inter-chain hydrogen-bonding network might be responsible for the low frequency of the hydroxyl stretching vibrations.^51^,^71^,^72^ DFT calculations also suggest the existence of such surface structures. Fig. 10 shows the computed Mn-rich OH terminated surface. By viewing the surface along the shows the computed Mn-rich OH terminated surface. By viewing the surface along the 〈100〉 axis two types of hydrogen can be seen to be present. Viewing the structure along the 〈010〉 direction reveals that both types are oriented such that they can participate in the hydrogen-bonding network, though to different degrees. The difference in hydrogen bonding manifests itself as differences in O⋯H (O–H) bond lengths, 2.23 Å (0.98 Å) and 1.92 Å (0.99 Å). Similarly, the calculated vibrational frequency of the OH stretch for these moieties differs slightly, 3518 cm100 shows the computed Mn-rich OH terminated surface. By viewing the surface along the 〈100〉 axis two types of hydrogen can be seen to be present. Viewing the structure along the 〈010〉 direction reveals that both types are oriented such that they can participate in the hydrogen-bonding network, though to different degrees. The difference in hydrogen bonding manifests itself as differences in O⋯H (O–H) bond lengths, 2.23 Å (0.98 Å) and 1.92 Å (0.99 Å). Similarly, the calculated vibrational frequency of the OH stretch for these moieties differs slightly, 3518 cm axis two types of hydrogen can be seen to be present. Viewing the structure along the shows the computed Mn-rich OH terminated surface. By viewing the surface along the 〈100〉 axis two types of hydrogen can be seen to be present. Viewing the structure along the 〈010〉 direction reveals that both types are oriented such that they can participate in the hydrogen-bonding network, though to different degrees. The difference in hydrogen bonding manifests itself as differences in O⋯H (O–H) bond lengths, 2.23 Å (0.98 Å) and 1.92 Å (0.99 Å). Similarly, the calculated vibrational frequency of the OH stretch for these moieties differs slightly, 3518 cm010 shows the computed Mn-rich OH terminated surface. By viewing the surface along the 〈100〉 axis two types of hydrogen can be seen to be present. Viewing the structure along the 〈010〉 direction reveals that both types are oriented such that they can participate in the hydrogen-bonding network, though to different degrees. The difference in hydrogen bonding manifests itself as differences in O⋯H (O–H) bond lengths, 2.23 Å (0.98 Å) and 1.92 Å (0.99 Å). Similarly, the calculated vibrational frequency of the OH stretch for these moieties differs slightly, 3518 cm direction reveals that both types are oriented such that they can participate in the hydrogen-bonding network, though to different degrees. The difference in hydrogen bonding manifests itself as differences in O···H (O–H) bond lengths, 2.23 Å (0.98 Å) and 1.92 Å (0.99 Å). Similarly, the calculated vibrational frequency of the OH stretch for these moieties differs slightly, 3518 cm^–1^ and 3464 cm^–1^. Both values, however, are in reasonable agreement with experiment (Fig. 9), suggesting that such a well-ordered inter-chain hydrogen-bonding network may explain the observed low frequency OH stretching modes.

In summary, the surface chemistry of the freshly prepared nano-structured catalysts can be described as follows. The near-surface region of the formed nanoparticles is more and more enriched in manganese with increasing OH^–^ concentration in the synthesis gel at the beginning of the hydrothermal synthesis of the catalysts. The accumulation of surface Mn species occurs during ageing in the autoclave resulting in catalysts with varying surface composition (Fig. 6, Table 2). The terminating Mn surface chains are composed of hydroxyl-hydrate species (Fig. 10), such as in Mn(OH)_2_·2H_2_O. Inter-chain hydrogen-bonding is crucial in terms of a stabilization of the Mn terminating layer on (010) surface planes.

Physisorbed water is essentially desorbed and OH groups are partially dehydroxylated during pre-treatment of the nano-structured MnWO_4_ catalysts in Ar at 673 K resulting in a surface that is enriched in oxygen vacancies, which can be shown by temperature-programmed oxidation (Fig. S4†). In these experiments, at first the catalysts were pretreated in argon at 673 K for 2 hours followed by TPO. The pretreatment corresponds to the pretreatment before catalysis. All catalysts exhibit a broad O_2_ consumption band centered at around 653 K indicating an oxygen-deficient surface after pretreatment in the inert gas that corresponds to the condition of the catalyst surface at *t* = 0 in the catalytic test. The surface concentration of oxygen vacancies within the present series of nano-structured MnWO_4_ catalysts is given in Table 2. The oxygen consumption is much smaller than the theoretical monolayer consumption. Therefore, the oxygen defects that react with gas phase oxygen are supposed to be localized on the surface. However, no direct correlation between oxygen defect density on the surface and synthesis conditions or the aspect ratio can be observed (Fig. S5†). The defect density increases with increasing pH and decreases again at the highest pH and in particular with increasing time due to defect healing processes. Generally, the more active catalysts exhibit in most of the cases a higher oxygen defect concentration after activation, but there is no clear correlation between activity and defect density (Fig. S5†).

**Fig. 11:**
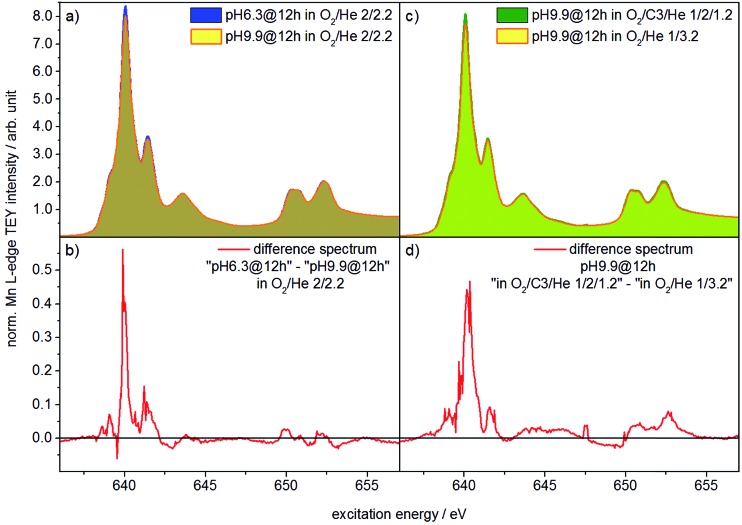
NEXAFS spectra measured at the Mn L_2,3_-edge in total electron yield (TEY) mode and a total pressure of 0.25 mbar in an oxidizing atmosphere (O_2_/He = 2/2.2 sccm) of (a) the catalysts pH 6.3@12 h and pH 9.9@12 h at *T* = 573 K; (b) shows the difference between the two spectra shown in (a); (c) presents the spectra of the catalyst pH 9.9@12 h in the feed (O_2_/C_3_H_8_/He = 1/2/1.2 sccm) and in an oxidizing atmosphere (O_2_/He = 2/2.2 sccm) at *T* = 653 K; the difference of the spectra shown in (c) is presented in (d).

Two catalysts that exhibit very different aspect ratios and activity (catalysts pH 6.3@12 h and pH 9.9@12 h) were studied by *in situ* X-ray absorption spectroscopy (Fig. 11). X-ray absorption at the Mn L edge is dominated by transition into Mn 3d states and, hence, characteristic of the Mn oxidation state and coordination.^73^ The Mn L-edge splits into two multiplets L_3_ at 638–642 eV and L_2_ at 650–655 eV due to spin–orbit splitting of the Mn 2p_3/2_ and 2p_1/2_ core levels. The oxidation state of manganese has a large influence on the peak position, line shape and intensity ratio of L_3_/L_2_ lines.^74^ Furthermore, coordination by different ligands, which has an impact on the ligand field strength and chemical bonding, causes variation in local symmetry and will change both line shape and intensity.^74^ The interpretation of Mn L-edge spectra is therefore not straightforward. However, generally, the L_3_/L_2_ white-line intensity ratio and the energy separation between the L_3_ and L_2_ peaks decrease with increasing valence of Mn.^75^ Moreover, the energy of the multiplet center of the Mn L-edge increases with increasing valence. Near edge X-ray absorption fine structure (NEXAFS) spectra at the Mn L_2,3_ edge at 573 K in the presence of flowing oxygen (Fig. 11a) indicate that the predominant oxidation state of Mn in both pH 6.3@12 h and pH 9.9@12 h catalysts is two.^73^ The applied measurement mode, total electron yield (TEY), while capturing surface information, has an information depth of several nanometers, making it sensitive to the near surface region. Therefore, the oxidation state 2+ is in agreement with the bulk oxidation state of crystalline, stoichiometric MnWO_4_.

Comparison of the spectra in Fig. 11a, in particular the difference between the spectra of the two catalysts (Fig. 11b), reveals, however, tiny differences that may indicate variations in the surface structure of the catalyst pH 6.3@12 h and catalyst pH 9.9@12 h. Because the spectrum of the catalyst pH 6.3@12 h exhibits more intensity at 639.6 eV, the L_3_/L_2_ edge intensity ratio of the latter is increased (Table S2†). Consequently, in the presence of oxygen the surface of the less active catalyst pH 6.3@12 h appears to be less oxidized compared to that of the catalyst pH 9.9@12 h that exhibits a higher propane consumption rate. The assignment is in agreement with the spectral changes observed by comparing the spectra of the catalyst in an oxygen atmosphere and in a propane oxidation feed (Fig. 11c and d). In the feed of the reactants the catalyst pH 9.9@12 h also exhibits higher intensity at 639.6 eV, *i.e.* a higher concentration of Mn^2+^ (or a higher degree of reduction), compared to that measured under an oxygen atmosphere.

These observations support that MnWO_4_ catalysts are subjected to changes in the valence state under conditions of propane oxidation and the catalyst operates as a redox-type catalyst with manganese being the redox active element. Similarly, the oxygen storage capability of Na_2_WO_4_/Mn/SiO_2_ applied as a catalyst in the oxidative coupling of methane has been attributed to the presence of manganese oxide.^76^

**Fig. 12:**
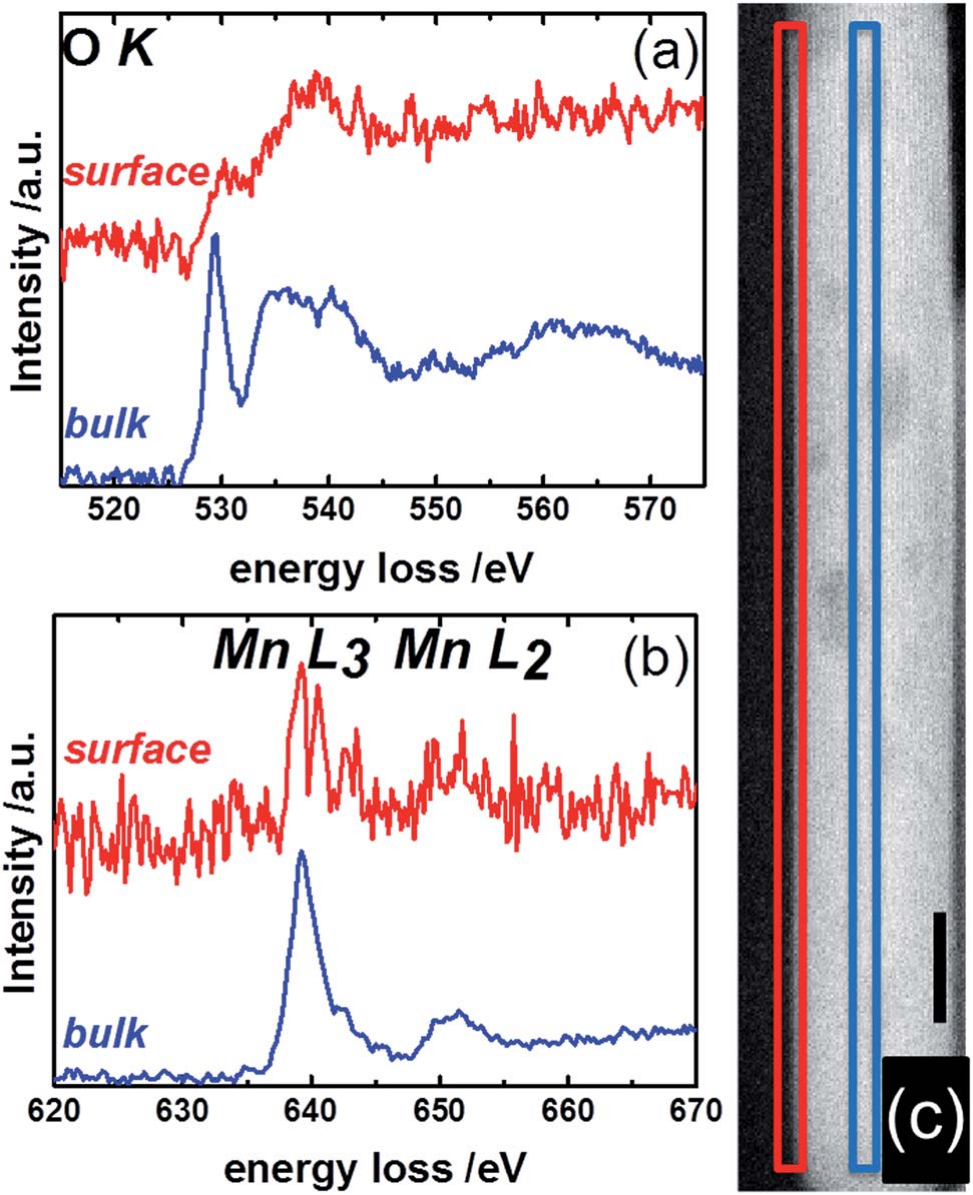
STEM-EELS measurements of the surface (red) and bulk (blue) of MnWO_4_ showing (a) the O K- and (b) the Mn L-edges. The squares in the STEM image of MnWO_4_ in (c) indicate the region where EELS measurements were conducted. Red: surface; blue: bulk. The black scale bare in (c) is 10 nm.

Complementary to NEXAFS, the surface and bulk of the catalyst pH 9.9@12 h were analyzed by line scans along the surface layer using electron energy loss spectroscopy (EELS) (Fig. 12). The O–K edge spectra of the bulk and surface differ in particular with respect to the peak at around 530 eV (Fig. 12a). The peak intensity is diminished in the surface spectrum. Taking into account the surface enrichment of Mn measured by XPS and Raman spectroscopy, the peak at 530 eV in the bulk spectrum might be dominated by contributions of oxygen atoms coordinated to tungsten.^77^ In contrast, the spectroscopic patterns on the oxygen K-edge in the surface spectrum resembles the O–K edge spectrum of a mixed Mn^2+^/Mn^3+^ oxide,^78^ confirming an enrichment of manganese on the surface and suggesting the presence of a Mn^2+^/Mn^3+^ redox couple in agreement with NEXAFS. The finding is supported by bulk and surface spectra on the Mn L-edge. The bulk spectrum corresponds mainly to Mn^2+^.^78^ Despite the low signal-to-noise ratio of the surface spectrum, a decrease in the L_3_/L_2_ white-line ratio is discernible in the surface spectrum compared to the bulk spectrum suggesting the presence of manganese also in an oxidation state higher than two on the surface of the catalyst.^78^,^79^

In summary, the nano-structured MnWO_4_ catalysts exhibit a clear enrichment in manganese on the surface as measured quantitatively by XPS and confirmed by Raman spectroscopy. The oxidation state of topmost manganese ions is rather low switching between 2+ and 3+ as suggested by NEXAFS and EELS.

**Scheme 1:**
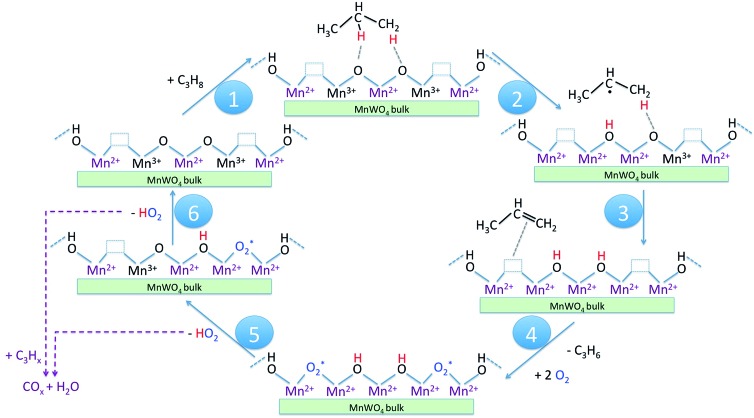
Catalytic cycle of the selective pathway in oxidative dehydrogenation of propane to propene on nano-structured MnWO_4_ catalysts. The reaction steps as discussed in the text are indicated by numbers in the blue circles.

In Scheme 1 the supposed catalytic cycle of the selective pathway in oxidative dehydrogenation of propane over MnWO_4_ is presented. Under reaction conditions in the presence of gas phase oxygen, the oxygen vacancies (Table 2, Fig. S5†) are partially filled, forming Mn–O–Mn sites on the outermost surface that represent appropriate adsorption sites for propane molecules (Scheme 1, Step 1).

**Fig. 13:**
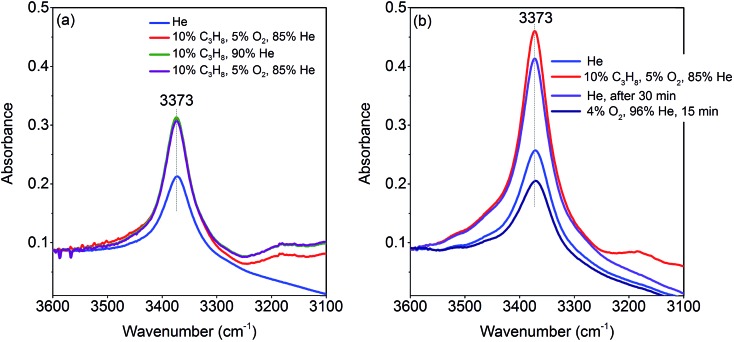
Operando FT-IR spectra of the catalyst pH 9.9@12 h at 673 K, gas composition and sequence of spectra as described in the legends from top to bottom; (a) flow rate 10 mL min^–1^; (b) flow rate 50 mL min^–1^.

The activation of the catalyst in inert gas does not lead to complete dehydroxylation, as evidenced by DRIFTS (Fig. 9) and confirmed by FTIR spectroscopy (Fig. 13) applied to analyze one of the most active catalysts (pH 9.9@12 h) under working conditions. After activation at 673 K in inert gas a sharp band due to residual Mn–OH groups at 3373 cm^–1^ is still present in the FTIR spectrum (Fig. 13a, blue spectrum) indicating that dehydroxylation is difficult. Therefore, residual OH groups have been included into the model of the freshly activated catalyst in Scheme 1 (sites at which Step 1 (propane adsorption) occurs).

The intensity of the band at 3373 cm^–1^ increases significantly after switching from He to the reaction gas feed at 673 K (Fig. 13a, red spectrum). The catalyst converts 2.6% propane and produces propene with a selectivity of 40.5% under the applied conditions in the FTIR cell as measured simultaneously by gas chromatography. The observed increase in the intensity of the peak assigned to Mn–O–H stretching vibrations is the result of establishing a steady state concentration of Mn–OH species under reaction conditions of the operando experiment due to abstraction of hydrogen from the C–H bonds of propane by nucleophilic surface Mn-oxygen species (Scheme 1, Steps 2 and 3) or due to hydroxylation by the reaction product water.

Steps 2 and 3 do not require the presence of oxygen in the reaction mixture. This could be experimentally verified by exposing the catalyst in a separate experiment to a 10% C_3_H_8_/90% He flow after removing all oxygen by flushing the cell with He for 90 minutes (Fig. 13a, green spectrum). The intensity of the OH band is increased to the same degree as it is in the oxygen-containing feed mixture. On switching back to C_3_H_8_/O_2_/He and thus starting the reaction, no further changes of the OH band are observed. This implies that the replenishment of OH groups is faster than dehydroxylation.

It can be shown that formation of OH groups is not simply caused by reaction of the catalyst surface with the reaction product water. The selectivity to CO_2_ and CO in the propane oxidation experiment in the FTIR cell corresponds to 37.2% and 6.7%, respectively. According to the reaction stoichiometry, the concentration of formed H_2_O comprises approximately 0.65 vol%. In a control experiment the catalyst was exposed to a flow of helium containing an even higher concentration of steam (1.2 vol% H_2_O) at the reaction temperature (Fig. S6†). The intensity of the hydroxyl peak increases only slightly demonstrating that the catalyst can abstract hydrogen more easily from propane than from H_2_O. Consequently, the increase in peak intensity under operation is not due to hydroxylation of the catalyst surface by the formed couple product water, but it is indeed due to abstraction of H atoms from the propane molecule by nucleophilic oxygen species associated with Mn on the catalyst surface (Scheme 1, Steps 2 and 3).

As a result of the homolytic splitting of the C–H bonds in propane the catalyst is more reduced after desorption of propene (Scheme 1, Step 4) and the catalytic cycle needs to comprise dehydroxylation and reoxidation steps to regenerate the catalyst. In classical redox mechanisms it is generally assumed that the surface is dehydroxylated first of all and the oxygen vacancies formed in this process are subsequently refilled by oxidation with gas-phase oxygen. To determine the sequence of dehydroxylation and reoxidation steps in the present catalytic cycle, the time evolution of the OH band intensity after switching from the reaction mixture (Fig. 13b, red spectrum) to He (Fig. 13b, violet spectrum) was monitored. In pure He the intensity declines only very slowly over a 30 minute period indicating that the OH groups formed by reaction of propane with the surface of MnWO_4_ cannot be dehydroxylated easily in inert gas at 673 K. Only after switching to a 10%O_2_/90%He mixture (Fig. 13b, dark blue spectrum) the peak area of the O–H band returns quickly to the area originally measured in He at 673 K (Fig. 13b, blue spectrum). The experiment would be in agreement with an oxygen-assisted dehydroxylation mechanism (Scheme 1, Steps 5 and 6) that can also be interpreted in terms of an oxidative dehydrogenation of active sites, which involves the intermediate formation of electrophilic intermediates, such as HO_2_ radicals.^80^,^81^ The assumption of intermediate formation of HO_2_ is highly speculative, but it could explain the limited selectivity of the MnWO_4_ catalysts to propene and the absence of oxygenates in the product mixture. Oxygen-containing reaction intermediates are most likely rapidly over-oxidized in the presence of such a reactive oxidizing agent.

## General discussion and conclusions

The most popular descriptor concerning the activity and selectivity of metal oxides in oxidation catalysis is the structural motif that is a component of crystalline bulk phases, although deviations from the regular bulk crystal structure on the surface of prominent oxidation catalysts have been clearly proven experimentally.^82^–^85^ The present series of nano-structured manganese tungstate catalysts is an impressive example that disproves again concepts solely based on ideal crystal structure motifs as functional active ensembles in oxidation catalysis, since all MnWO_4_ catalysts in the series under study exhibit the same bulk crystal structure and bulk chemical composition and are phase pure and homogenous, but show very different catalytic properties.

Only the needle-like anisotropy of the primary catalyst particles is developed differently within the sample series; however, a direct correlation between the shape and catalytic properties was not observed. However, we discovered that MnWO_4_ evolves selectivity when a layer of MnO_*x*_ is exposed on the surface, which occurs preferentially on the (010) plane.^50^ In the present case of hydrothermally prepared nano-structured manganese tungstate catalysts the unique surface layer is established under the hydrothermal preparation conditions. Synthesis parameters, such as pH and ageing time, can be used to control the formation of the surface layer as evidenced by multi-wavelength Raman spectroscopy (Fig. 8). Consequently, the catalytic performance is governed by the synthesis to a certain degree (Fig. 3).

However, the catalysts undergo further surface changes under reaction conditions of propane oxidation. No bulk structural modifications were observed with time on stream. The catalysts prepared at pH < 9 just deactivate slightly, but catalysts prepared at pH > 9 show a more complex behavior including the initial activation followed by deactivation or just an activation process. The higher complexity in the time-on-stream behavior of the group of catalysts prepared at pH > 9 is attributed to the basic conditions in the autoclave and prolonged synthesis times that lead to more severe dissolution–recrystallization processes compared to the synthesis at lower pH and shorter times. Extended defects are formed that undergo changes under reaction conditions as evidenced by electron microscopy (Fig. 5).

Photoelectron spectroscopy of fresh and used catalysts revealed a clear correlation between propane consumption rates and the concentration of surface Mn ions in the near-surface region of the well-defined nano-structured manganese tungstate catalysts (Fig. 6). Therefore, the catalytic activity is attributed to manganese oxide surface species that have also been detected qualitatively by Raman spectroscopy (Fig. 7 and 8) and EELS (Fig. 12). Decoupling of the electronic structure of these manganese oxide surface species from the bulk has been proven by the observation of resonance effects measured by multi-wavelength Raman spectroscopy (Fig. 8).

NEXAFS spectroscopy suggested that the activation of propane on MnWO_4_ occurs according to a redox mechanism that involves a change in the formal oxidation state of Mn. So far, a Mn^3+^/Mn^2+^ redox couple is proposed. NEXAFS spectroscopy was performed at reaction temperature and in the presence of the flowing reactants, albeit at reduced pressure. The *in situ* NEXAFS study of two catalysts that differ significantly in steady-state confirmed the general trend in the differences between the two catalysts in particular with respect to the degree of oxidation (Fig. 11). It should be noted at this point that NEXAFS spectroscopy under operating conditions illustrates the experimental challenge that is involved in the detection of differences within a surface layer of a thickness of one or few nanometers in the presence of a major bulk phase composed of the same chemical elements.

Operando infrared spectroscopy clearly confirms that hydrogen atoms are abstracted from the propane molecule by surface manganese oxygen species under the formation of surface hydroxyl groups (Fig. 13a). Furthermore, infrared spectroscopy suggests oxidative dehydrogenation of active sites instead of a classical dehydroxylation-re-oxidation mechanism for the regeneration of the catalyst (Fig. 13b). We postulate that such a mechanism might include intermediate formation of HO_2_ radicals, which could be responsible for deep oxidation to carbon oxides observed as the major side products on this type of catalyst. Direct experimental proof of these intermediates is challenging and requires further studies. The infrared spectroscopy result still suggests preferential occurrence of Mn^2+^/Mn^3+^ single sites on the surface of the catalyst that do not allow facile four-electron reduction of molecular oxygen other than on assemblies of four redox active metal atoms in one surface cluster.^86^,^87^

The present study demonstrates that the formation of a thin layer of active transition metal oxide species on the surface of a well-defined crystalline bulk structure may provide a tool for tuning catalytic performance in alkane oxidation reactions. However, it illustrates at the same time the constraints in the discovery of catalysts with improved performance by big data analysis or other descriptor-based approaches when just bulk structural parameters are taken into account. We postulate that efficient catalysts for lower alkane activation might be found based on nano-structuring of crystalline materials that have shown so far no catalytic activity in their macro-structural appearance. Hydrothermal synthesis^51^,^88^,^89^ or atomic layer deposition^90^ might be a promising synthetic tool in this respect. Another descriptor might be the surface lattice oxygen bonding energy that has an impact on the mechanism of catalyst regeneration and the abundance of highly reactive electrophilic intermediates on the surface of the catalyst under reaction conditions that over-oxidize desired products including olefins and oxygenates to carbon oxides.

## Conflicts of interest

There are no conflicts to declare.

## Supplementary Material

Supplementary information

## References

[cit1] Schwarz H., Shaik S., Li J. (2017). J. Am. Chem. Soc..

[cit2] Coperet C. (2010). Chem. Rev..

[cit3] Cheng M.-J., Goddard W. A. (2015). J. Am. Chem. Soc..

[cit4] Cheng M.-J., Goddard W. A. (2013). J. Am. Chem. Soc..

[cit5] Zhu Y., Sushko P. V., Melzer D., Jensen E., Kovarik L., Ophus C., Sanchez-Sanchez M., Lercher J. A., Browning N. D. (2017). J. Am. Chem. Soc..

[cit6] Melzer D., Xu P., Hartmann D., Zhu Y., Browning N. D., Sanchez-Sanchez M., Lercher J. A. (2016). Angew. Chem., Int. Ed..

[cit7] Shiju N. R., Liang X., Weimer A. W., Liang C., Dai S., Guliants V. V. (2008). J. Am. Chem. Soc..

[cit8] Somorjai G. A., Park J. Y. (2008). Angew. Chem., Int. Ed..

[cit9] Schlögl R. (2015). Angew. Chem., Int. Ed..

[cit10] Van Santen R. A. (2009). Acc. Chem. Res..

[cit11] Kung H. H., Pellet R. J., Burwell R. L. (1976). J. Am. Chem. Soc..

[cit12] Yang N., Medford A. J., Liu X., Studt F., Bligaard T., Bent S. F., Nørskov J. K. (2016). J. Am. Chem. Soc..

[cit13] Hansen P. L., Wagner J. B., Helveg S., Rostrup-Nielsen J. R., Clausen B. S., Topsøe H. (2002). Science.

[cit14] Zhou K., Li Y. (2012). Angew. Chem., Int. Ed..

[cit15] Haruta M. (2003). Chem. Rec..

[cit16] Li Y., Shen W. (2014). Chem. Soc. Rev..

[cit17] Volta J.-C. (2001). Top. Catal..

[cit18] Volta J. C., Portefaix J. L. (1985). Appl. Catal..

[cit19] Tatibouet J. M., Germain J. E., Volta J. C. (1983). J. Catal..

[cit20] Smith M. R., Ozkan U. S. (1993). J. Catal..

[cit21] Hargreaves J. S. J., Hutchings G. J., Joyner R. W., Kiely C. J. (1992). J. Catal..

[cit22] Schwach P., Frandsen W., Willinger M.-G., Schlögl R., Trunschke A. (2015). J. Catal..

[cit23] Schwach P., Hamilton N., Eichelbaum M., Thum L., Lunkenbein T., Schlögl R., Trunschke A. (2015). J. Catal..

[cit24] Hu L., Peng Q., Li Y. (2008). J. Am. Chem. Soc..

[cit25] Xie X., Li Y., Liu Z.-Q., Haruta M., Shen W. (2009). Nature.

[cit26] Mai H.-X., Sun L.-D., Zhang Y.-W., Si R., Feng W., Zhang H.-P., Liu H.-C., Yan C.-H. (2005). J. Phys. Chem. B.

[cit27] Zhou K., Wang X., Sun X., Peng Q., Li Y. (2005). J. Catal..

[cit28] Wu Z., Li M., Overbury S. H. (2012). J. Catal..

[cit29] Hua Q., Cao T., Gu X.-K., Lu J., Jiang Z., Pan X., Luo L., Li W.-X., Huang W. (2014). Angew. Chem., Int. Ed..

[cit30] Grasselli R. K., Buttrey D. J., DeSanto P., Burrington J. D., Lugmair C. G., Volpe Jr A. F., Weingand T. (2004). Catal. Today.

[cit31] Noguera C., Goniakowski J. (2013). Chem. Rev..

[cit32] Chang C.-R., Huang Z.-Q., Li J. (2016). Wiley Interdiscip. Rev.: Comput. Mol. Sci..

[cit33] Liu X., Zhou K., Wang L., Wang B., Li Y. (2009). J. Am. Chem. Soc..

[cit34] Wu Z., Li M., Howe J., Meyer H. M., Overbury S. H. (2010). Langmuir.

[cit35] Paier J., Penschke C., Sauer J. (2013). Chem. Rev..

[cit36] Hävecker M., Wrabetz S., Kröhnert J., Csepei L.-I., Naumann d'Alnoncourt R., Kolen’ko Y. V., Girgsdies F., Schlögl R., Trunschke A. (2012). J. Catal..

[cit37] Hävecker M., Mayer R. W., Knop-Gericke A., Bluhm H., Kleimenov E., Liskowski A., Su D., Follath R., Requejo F. G., Ogletree D. F., Salmeron M., Lopez-Sanchez J. A., Bartley J. K., Hutchings G. J., Schlögl R. (2003). J. Phys. Chem. B.

[cit38] Eichelbaum M., Glaum R., Hävecker M., Wittich K., Heine C., Schwarz H., Dobner C.-K., Welker-Nieuwoudt C., Trunschke A., Schlögl R. (2013). ChemCatChem.

[cit39] Heine C., Hävecker M., Sanchez-Sanchez M., Trunschke A., Schlögl R., Eichelbaum M. (2013). J. Phys. Chem. C.

[cit40] Heine C., Hävecker M., Stotz E., Rosowski F., Knop-Gericke A., Trunschke A., Eichelbaum M., Schlögl R. (2014). J. Phys. Chem. C.

[cit41] Eichelbaum M., Hävecker M., Heine C., Wernbacher A. M., Rosowski F., Trunschke A., Schlögl R. (2015). Angew. Chem., Int. Ed..

[cit42] Heine C., Havecker M., Trunschke A., Schlogl R., Eichelbaum M. (2015). Phys. Chem. Chem. Phys..

[cit43] Stern D. L., Grasselli R. K. (1997). J. Catal..

[cit44] Mazzocchia C., Aboumrad C., Diagne C., Tempesti E., Herrmann J. M., Thomas G. (1991). Catal. Lett..

[cit45] Cadus L. E., Ferretti O. (2002). Appl. Catal., A.

[cit46] Lezla O., Bordes E., Courtine P., Hecquet G. (1997). J. Catal..

[cit47] Vie D., Martínez E., Sapiña F., Folgado J.-V., Beltrán A., Valenzuela R. X., Cortés-Corberán V. (2004). Chem. Mater..

[cit48] Stern D. L., Grasselli R. K. (1997). J. Catal..

[cit49] Salamanca M., Licea Y. E., Echavarria A., Faro Jr A. C., Palacio L. A. (2009). Phys. Chem. Chem. Phys..

[cit50] Li X., Lunkenbein T., Pfeifer V., Jastak M., Nielsen P. K., Girgsdies F., Knop-Gericke A., Rosowski F., Schlögl R., Trunschke A. (2016). Angew. Chem., Int. Ed..

[cit51] Li X., Lunkenbein T., Krohnert J., Pfeifer V., Girgsdies F., Rosowski F., Schlogl R., Trunschke A. (2016). Faraday Discuss..

[cit52] Callahan J. L., Grasselli R. K. (1963). AIChE J..

[cit53] Ghiringhelli L. M., Vybiral J., Levchenko S. V., Draxl C., Scheffler M. (2015). Phys. Rev. Lett..

[cit54] Yu S. H., Liu B., Mo M. S., Huang J. H., Liu X. M., Qian Y. T. (2003). Adv. Funct. Mater..

[cit55] Yeh J. J., Lindau I. (1985). At. Data Nucl. Data Tables.

[cit56] Knop-Gericke A., Kleimenov E., Hävecker M., Blume R., Teschner D., Zafeiratos S., Schlögl R., Bukhtiyarov V. I., Kaichev V. V., Prosvirin I. P., Nizovskii A. I., Bluhm H., Barinov A., Dudin P. and Kiskinova M., in Advances in Catalysis, ed. C. G. Bruce and K. Helmut, Academic Press, 2009, vol. 52, pp. 213–272.

[cit57] Giannozzi P., Baroni S., Bonini N., Calandra M., Car R., Cavazzoni C., Ceresoli D., Chiarotti L. G., Cococcioni M., Dabo I., Corso A. D., Gironcoli S. d., Fabris S., Fratesi G., Gebauer R., Gerstmann U., Gougoussis C., Kokalj A., Lazzeri M., Martin-Samos L., Marzari N., Mauri F., Mazzarello R., Paolini S., Pasquarello A., Paulatto L., Sbraccia C., Scandolo S., Sclauzero G., Seitsonen P. A., Smogunov A., Umari P., Wentzcovitch M. R. (2009). J. Phys.: Condens. Matter.

[cit58] Perdew J. P., Burke K., Ernzerhof M. (1996). Phys. Rev. Lett..

[cit59] Cococcioni M., de Gironcoli S. (2005). Phys. Rev. B: Condens. Matter Mater. Phys..

[cit60] Macavei J., Schulz H. (1993). Zeitschrift für Kristallographie – Crystalline Materials.

[cit61] Marzari N., Vanderbilt D., De Vita A., Payne M. C. (1999). Phys. Rev. Lett..

[cit62] Keeling R. (1957). Acta Crystallogr..

[cit63] Iliev M. N., Gospodinov M. M., Litvinchuk A. P. (2009). Phys. Rev. B: Condens. Matter Mater. Phys..

[cit64] Choi W. S., Taniguchi K., Moon S. J., Seo S. S. A., Arima T., Hoang H., Yang I.-S., Noh T. W., Lee Y. S. (2010). Phys. Rev. B: Condens. Matter Mater. Phys..

[cit65] Buciuman F., Patcas F., Craciun R., Zahn D. R. T. (1999). Phys. Chem. Chem. Phys..

[cit66] Malavasi L., Galinetto P., Mozzati M. C., Azzoni C. B., Flor G. (2002). Phys. Chem. Chem. Phys..

[cit67] Kim H., Kosuda K. M., Van Duyne R. P., Stair P. C. (2010). Chem. Soc. Rev..

[cit68] Moskvin A. S. (2002). Phys. Rev. B: Condens. Matter Mater. Phys..

[cit69] Atanasov M., Brunold T. C., Güdel H. U., Daul C. (1998). Inorg. Chem..

[cit70] Kanan S. M., Lu Z., Cox J. K., Bernhardt G., Tripp C. P. (2002). Langmuir.

[cit71] Henderson M. A. (2002). Surf. Sci. Rep..

[cit72] Salah M. B., Vilminot S., Mhiri T., Kurmoo M. (2004). Eur. J. Inorg. Chem..

[cit73] Gilbert B., Frazer B. H., Belz A., Conrad P. G., Nealson K. H., Haskel D., Lang J. C., Srajer G., De Stasio G. (2003). J. Phys. Chem. A.

[cit74] Cramer S. P., DeGroot F. M. F., Ma Y., Chen C. T., Sette F., Kipke C. A., Eichhorn D. M., Chan M. K., Armstrong W. H. (1991). J. Am. Chem. Soc..

[cit75] Qiao R., Chin T., Harris S. J., Yan S., Yang W. (2013). Curr. Appl. Phys..

[cit76] Fleischer V., Littlewood P., Parishan S., Schomäcker R. (2016). Chem. Eng. J..

[cit77] Kielwein M., Saiki K., Roth G., Fink J., Paasch G., Egdell R. G. (1995). Phys. Rev. B: Condens. Matter Mater. Phys..

[cit78] Tan H., Verbeeck J., Abakumov A., Van Tendeloo G. (2012). Ultramicroscopy.

[cit79] Riedl T., Gemming T., Wetzig K. (2006). Ultramicroscopy.

[cit80] Lomonosov V. I., Sinev M. Y. (2016). Kinet. Catal..

[cit81] Haber J., Turek W. (2000). J. Catal..

[cit82] Centi G. (1993). Catal. Today.

[cit83] Kleimenov E., Bluhm H., Haevecker M., Knop-Gericke A., Pestryakov A., Teschner D., Lopez-Sanchez J. A., Bartley J. K., Hutchings G. J., Schloegl R. (2005). Surf. Sci..

[cit84] Celaya Sanfiz A., Hansen T. W., Teschner D., Schnörch P., Girgsdies F., Trunschke A., Schlögl R., Looi M. H., Hamid S. B. A. (2010). J. Phys. Chem. C.

[cit85] Brookes C., Bowker M., Wells P. (2016). Catalysts.

[cit86] Schlögl R. (2011). Top. Catal..

[cit87] Maganas D., Trunschke A., Schlögl R., Neese F. (2016). Faraday Discuss..

[cit88] Sanchez Sanchez M., Girgsdies F., Jastak M., Kube P., Schlögl R., Trunschke A. (2012). Angew. Chem., Int. Ed..

[cit89] Noack J., Rosowski F., Schlögl R., Trunschke A. (2014). Z. Anorg. Allg. Chem..

[cit90] Strempel V. E., Löffler D., Kröhnert J., Skorupska K., Johnson B., d'Alnoncourt R. N., Driess M., Rosowski F. (2016). J. Vac. Sci. Technol., A.

