# Supplementary material for: How to control selectivity in alkane oxidation?
Source: Chem Sci. 2018 Dec 20;10(8):2429–43. doi: 10.1039/c8sc04641g (PMC6385647; doi:10.1039/c8sc04641g)
Supplement: Supplementary file 1 [file SC-010-C8SC04641G-s001.pdf]

Electronic Supplementary Material (ESI) for Chemical Science.
This journal is © The Royal Society of Chemistry 2018

Supporting Information

How to control selectivity in alkane oxidation?

Xuan Li,[1,2] *Detre Teschner,*[1,3] *Verena Streibel,*[1] *Thomas Lunkenbein,*[1] *Liudmyla Masliuk,*[1] *Teng Fu,*[1] *Yuanqing Wang,*[1,2] *Travis Jones,*[1] *Friedrich Seitz,*[1] *Frank Girgsdies,*[1] *Frank Rosowski,*[2,4] *Robert Schlögl,*[1,3] *Annette Trunschke*[1,*]

[1]Department of Inorganic Chemistry, Fritz-Haber-Institut der Max-Planck-Gesellschaft, Faradayweg 4-6, 14195 Berlin, Germany

[2]UniCat-BASF Joint Lab, Technische Universität Berlin, Sekr. EW K 01, Hardenbergstraße 36, 10623 Berlin, Germany

[3]Department of Heterogeneous Reactions, Max-Planck-Institut für Chemische Energiekonversion, Stiftstraße 34-36, 45470 Mülheim a. d. Ruhr, Germany

[4]BASF SE, Process Research and Chemical Engineering, Heterogeneous Catalysis, Carl-Bosch-Straße 38, 67056 Ludwigshafen, Germany

kobelixnju@gmail.com
teschner@fhi-berlin.mpg.de
streibel@stanford.edu
lunkenbein@fhi-berlin.mpg.de
liumasliuk@fhi-berlin.mpg.de
teng@fhi-berlin.mpg.de
yqwang@fhi-berlin.mpg.de
trjones@fhi-berlin.mpg.de
seitzfr@fhi-berlin.mpg.de
girgsdie@fhi-berlin.mpg.de
frank.rosowski@basf.com
acsek@fhi-berlin.mpg.de
trunschke@fhi-berlin.mpg.de

Supporting information

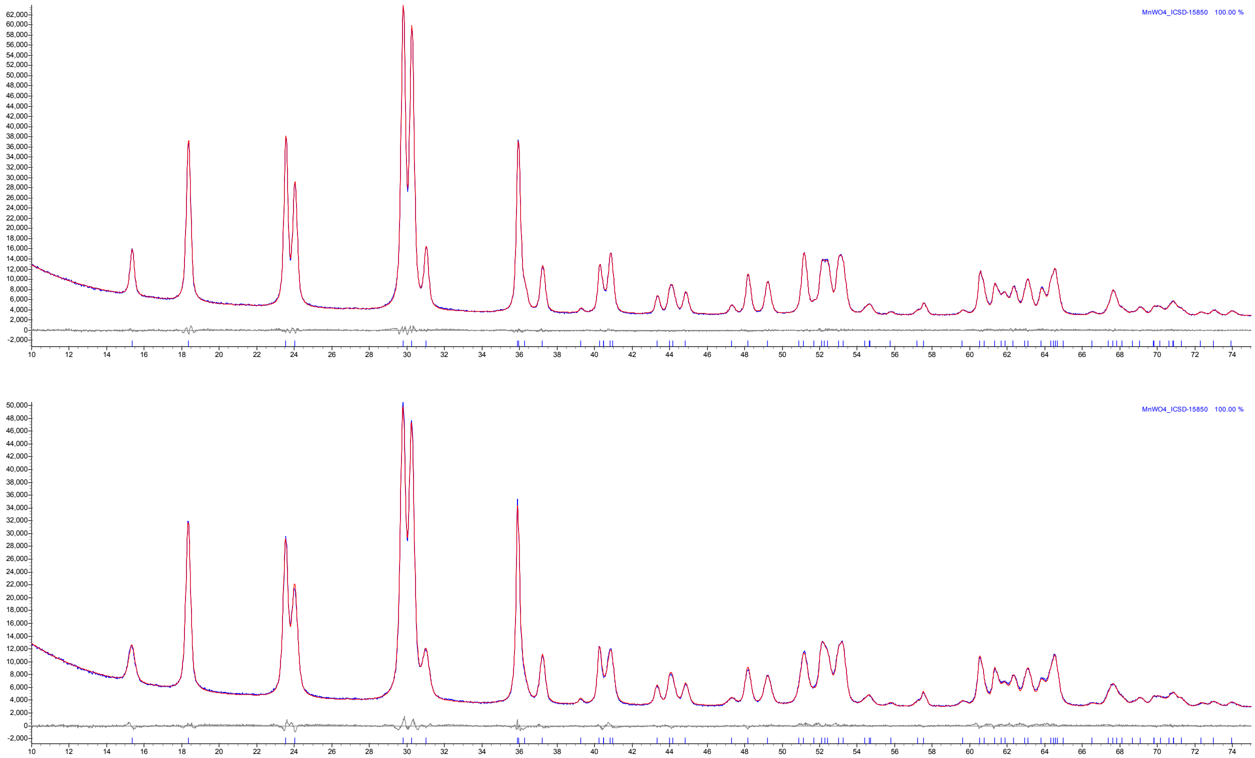

Figure S1. XRD patterns and results of Rietveld refinement exemplarily shown for pH6.3@12h with short rod-like particles (AR=1.5) (top) and phH9.9@12h with elongated rod-like particles (AR=5.1) (bottom).

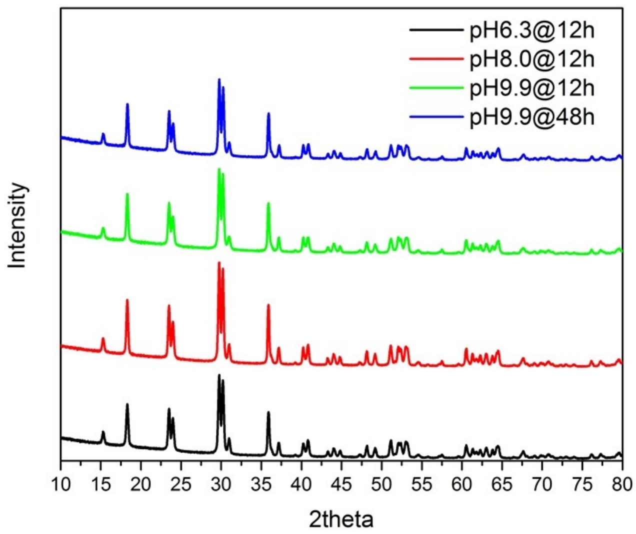

Figure S2. XRD patterns of used catalysts (see Table S1 for results of refinement).

Table S1. Crystallite size and aspect ratio calculated from anisotropic fitting in Rietveld refinement of the XRD patterns

	as synthesized					after catalysis				
	crystallite size			aspect ratio		crystallite size			aspect ratio	
	D_a [nm]	D_b [nm]	D_c [nm]	D_a/D_b	D_c/D_b	D_a [nm]	D_b [nm]	D_c [nm]	D_a/D_b	D_c/D_b
pH6.3@12h	27.5	28.1	40.8	1.0	1.5	27.7	26.9	35.2	1.0	1.3
pH6.7@12h	27.8	27.1	46.2	1.0	1.7					
pH8.0@12h	28.7	25.4	66.1	1.1	2.6	31.7	26.8	45.9	1.2	1.7
pH9.1@12h	26.6	21.3	53.6	1.2	2.5					
pH9.9@12h	23.9	17.5	58.1	1.4	3.3	29.5	20.9	41.2	1.4	2.0
pH9.9@24h	22.5	18.6	49.9	1.2	2.7					
pH9.9@48h	23.3	21.8	47.5	1.1	2.2	30.7	24.9	41.6	1.2	1.7

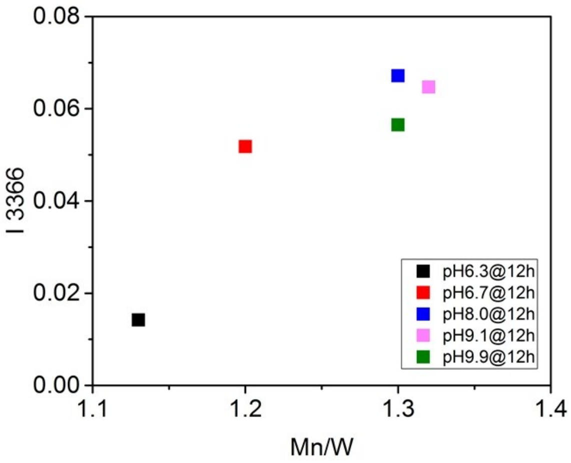

Figure S3. Intensity of the main peak at 3366 cm^{-1} in the infrared spectra in Figure 9 (main text) as a function of the Mn/W ratio determined by XPS (Table 2 in the main text).

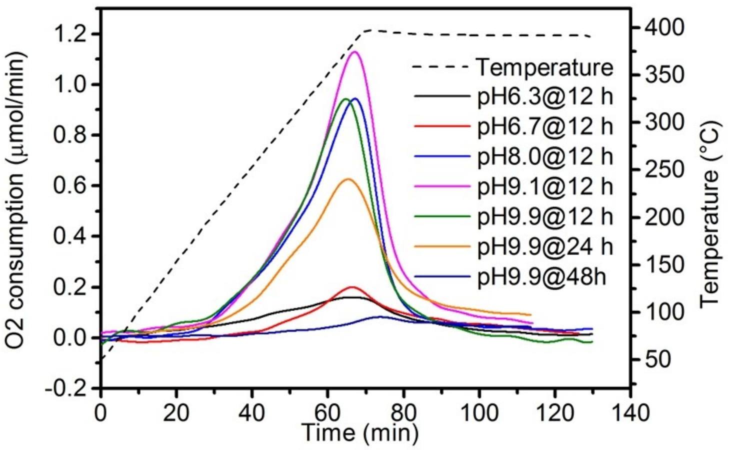

Figure S4. Profiles of temperature-programmed oxidation after thermal pre-treatment of the catalysts in Ar at T=673 K.

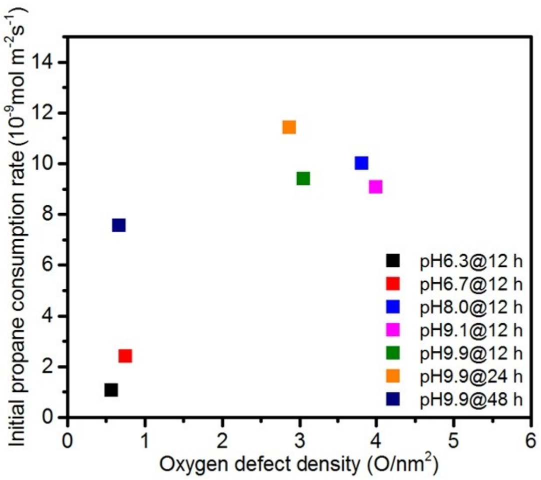

Figure S5. Initial consumption rate of propane as a function of oxygen defect concentration measured by temperature-programmed oxidation (Figure S4, Table 2 in the main text).

Table S2. Intensity of NEXAFS Mn $L_{2,3}$ edges in TEY mode (Fig. 12 in the main text)

Intensity /a.u.	I L_3 at 640.0 eV	I L_2 at 652.3 eV	Branching ratio of L_3/L_2
pH6.3@12h	8.39	2.03	4.13
pH9.9@12h	8.03	2.02	3.98

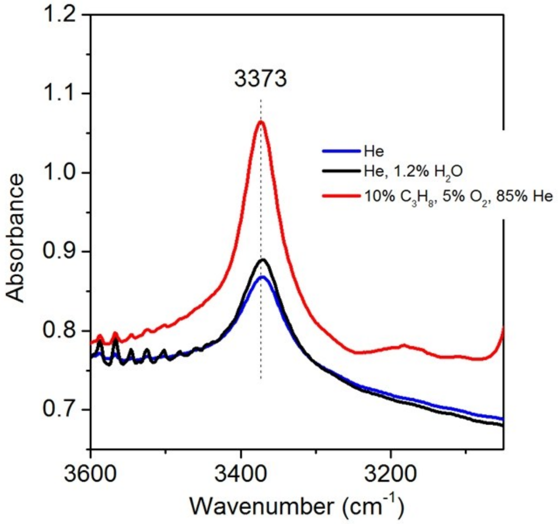

Figure S6. FT-IR spectra of the catalyst pH9.9@12h at 673 K at a flow rate of 5 ml/min, temperature of 673 K and gas composition as described in the legend.
